# Impaired suppressive effect of FoxP3 regulatory T cells on B cells in multiple sclerosis

**DOI:** 10.1186/s12974-026-03776-5

**Published:** 2026-05-02

**Authors:** Viktoria B. Greeck, Cornelia Würthwein, Karina Mimura, Katharina Mattes, Michael Kutza, Lucas Schirmer, Richard Fairless, Sarah K. Williams, Sven Jarius, Jürgen Haas, Klemens Ruprecht, Brigitte Wildemann

**Affiliations:** 1 Division of Neuroimmunology, Department of Neurology, Heidelberg University Hospital, Heidelberg, Germany; 2 Interdisciplinary Center for Neurosciences (IZN), Heidelberg University, Heidelberg, Germany; 3 Clinical Cooperation Unit (CCU) Neurooncology, German Cancer Consortium (DKTK), German Cancer Research Center (DKFZ), Heidelberg, Germany; 4 Division of Neuroimmunology, Department of Neurology, Medical Faculty Mannheim, Heidelberg University, Mannheim, Germany; 5 Mannheim Center for Translational Neuroscience (MCTN) and Institute for Innate Immunoscience (MI3), Medical Faculty Mannheim, Heidelberg University, Mannheim, Germany

**Keywords:** B cells, Regulatory T cells (Tregs), Multiple sclerosis, Healthy donors, Proliferation, Interleukin 6, APC markers, Calcium signaling, Transcriptome, NF-κB, NFATc1, Single-cell analysis, B cell receptor

## Abstract

**Background:**

B cells are key contributors to the pathogenesis of many autoimmune diseases (AID), including multiple sclerosis (MS), and appear to evade the peripheral tolerance checkpoints that normally maintain immune homeostasis. The fate of B cells at these checkpoints is believed to be regulated by intracellular Ca^2+^ signaling cascades triggered through engagement of B cell receptors (BCR), and by the suppressive effects of regulatory T cells (Tregs). However, most of the current knowledge about Treg–B cell interaction comes from animal studies, while data from human studies, particularly in the context of AID, are sparse. In contrast, impaired Treg-mediated inhibition of conventional T cells (Tcons) has already been described for several AID, including MS.

**Objective:**

To assess the ability of Tregs to suppress activated B cells in healthy individuals and patients with MS.

**Methods:**

B and T cell populations were isolated from 40 MS patients and 98 age- and sex-matched healthy donors (HD). Single-cell live Ca²⁺ imaging was used to assess early activation signals in B cells. In vitro proliferation assays and coculture experiments were employed to evaluate downstream responses, including proliferation, transcription factor activation (NFATc1, NF-ĸB), interleukin 6 (IL-6) release, and surface expression levels of antigen-presenting capacity (APC) markers both in anti-IgM/anti-CD40-stimulated B cells alone, and in the presence of Tregs.

**Results:**

We demonstrate that Tregs exert a robust suppressive effect on B cell proliferation, IL-6 secretion and NFATc1 which is [1] independent of Ca^2+^ signaling [2], dependent on direct cell contact, and [3] impaired in MS. In contrast, early Ca^2+^ responses and downstream effects of anti-IgM/anti-CD40 stimulation, including activation of NFATc1 and NF-κB, as well as proliferation, did not differ between MS- and HD-derived B cells.

**Conclusion:**

This study provides new data on Treg-mediated suppression of B cells in humans, including at single-cell level. Our findings show that the Treg dysfunction in MS previously described in the context of Tcon regulation extends to B cell regulation. Given the critical role of B cells in MS pathogenesis, this impaired Treg–B cell interaction may represent a previously underappreciated disease mechanism with potentially important therapeutic implications.

**Supplementary Information:**

The online version contains supplementary material available at 10.1186/s12974-026-03776-5.

## Introduction

FoxP3-regulatory T cells (Tregs) are known to downregulate the activation and proliferation of conventional T cells (Tcons). However, data on Treg–B cell interaction in humans remains limited [1, 2], and our current understanding of Treg-mediated suppression of B cells is largely based on animal studies. These studies demonstrate that CD4^+^CD25^+^ Tregs can suppress B cells in various ways, including direct suppression of B cell proliferation and immunoglobulin production in vitro, partly via contact-dependent mechanisms [3–5]. Murine Tregs have also been shown to modulate B cell activation via immune checkpoint pathways [6, 7] and restrain the antigen-presenting function of B cells [8]. Furthermore, follicular regulatory T cells (TFr cells) can limit B cell responses in germinal centers (GCs) [9–13]. Indirect evidence that Tregs play an important role in preventing excessive B cell responses comes from studies with FoxP3-deficient mice. The absence of functional Tregs in these mice led to increased GC responses, increased serum immunoglobulin levels, accumulation of autoreactive B cells, and broad autoantibody production [13, 14]. Although impaired inhibitory function of Tregs on T lymphocytes has been described in several autoimmune diseases [15], it remains unclear whether similar deficits apply to Treg-mediated regulation of B cells. Multiple sclerosis (MS) is a prototypic autoimmune disorder that serves as a key model for studying the role of Tregs in autoimmunity. Moreover, self-reactive B cells play a central pathogenic role in MS, as evidenced by the clinical benefit of B cell-depleting therapies, which reduce relapse rates and the formation of new brain lesions in patients with relapsing‒remitting MS [16–21]. Similar to systemic lupus erythematosus (SLE) and rheumatoid arthritis (RA), ineffective selection against autoreactive B cell clones at peripheral (but not central) immune checkpoints has been suggested to underly B cell-mediated autoimmunity in MS [22]. However, the exact mechanisms by which pathogenic B cells escape tolerance checkpoints that maintain immune homeostasis under normal physiological conditions are still widely unknown. Unraveling these mechanisms is essential for the understanding of MS pathophysiology and B cell-related autoimmunity in general. During all stages of their maturation, B cells integrate signals from the B cell receptor (BCR) with those from costimulatory receptors such as CD40, B cell-activating factor receptor (BAFF), and toll-like receptors (TLRs), which collectively modulate B cell survival, metabolism, and proliferation [23–26]. The fate of developing B cells—particularly their deletion at tolerance checkpoints—is governed by intracellular Ca^2+^ signaling. Strong BCR engagement by self-antigens induces pro-apoptotic transcriptional programs that eliminate autoreactive B cell clones via Ca^2+^-dependent pathways, while tonic signaling promotes cell survival [26–33].

Impaired peripheral tolerance has been linked to reduced suppression by Tregs expressing the lineage-specific transcription factor forkhead box protein 3 (FoxP3) [34]. Alterations in the Treg compartment have repeatedly been demonstrated in human autoimmunity [15]. In MS, these include changes in Treg numbers, altered subpopulation distribution, and diminished suppressive capacity [35–41]. Notably, MS patient-derived Tregs fail to efficiently suppress Ca^2+^ influx in Tcons, resulting in heightened T cell activation, proliferation, and release of proinflammatory cytokines [42].

In the present study, we chose a combined anti-IgM/anti-CD40 stimulation to activate B cells in vitro. Crosslinking of the BCR via surface IgM mimics antigen engagement and provides the primary activation signal, whereas ligation of CD40 by CD40L on activated CD4^+^ T cells delivers a non-redundant “second signal” that is essential for full B cell proliferation, survival, class-switch recombination and GCs [43–45]. Genetic disruption of CD40 or CD40L in mice and humans leads to profound defects in T cell-dependent antibody responses and strongly reduced GC reactions, underscoring the central role of CD40-CD40L interactions in physiological B cell activation [46]. In vitro, simultaneous BCR and CD40 engagement synergistically augments Ca^²+^ influx, NFAT and NF-kB activation, and entry into the cell cycle compared with either stimulus alone, and is therefore widely used as a reductionist model of T cell-dependent B cell activation [26, 43, 44]. Against this background, anti-IgM/anti-CD40 costimulation represents an experimentally tractable approximation of cognate T cell help in GCs and provides a suitable platform to study how Tregs modulate B cell signaling and proliferation.

### Patients

The study cohort included 40 patients with relapsing–remitting MS (MS; median age 31 years; range 19–55; 31 female) diagnosed according to the revised 2017 McDonald criteria [47] and 98 healthy donors (HD; median age 29 years, range 21–57; 65 female). Patients were recruited at the Department of Neurology, Heidelberg University Hospital, Germany. Among MS patients, the median disease duration was 1.5 years (range 0–11), with a median Expanded Disability Status Scale (EDSS) score of 2.0 (range 1.0–3.5), and a history of 1–3 (median 1) attacks. At the time of blood sampling, 24 patients had clinically active disease, while 16 were in remission. None of the patients included in the functional analyses were receiving disease-modifying therapy at the time of blood sampling; furthermore, none had received steroids or immunosuppressive treatment within the preceding 3 months. The study was approved by the ethics committee of Heidelberg University Hospital, and written informed consent was obtained from all participants. Demographic and clinical data of all MS patients and sex- and age matched HDs are summarized in Table 1, and listed for each study subject in Supplementary Table 3.

**Table 1:** Summary of the demographic and clinical data of MS patients and age- and sex- matched heathy control donors

	Total		Proliferation assay		Crisscross		Interleukin 6		Ca^2+^ Imaging	
	MS	HD	MS	HD	MS	HD	MS	HD	MS	HD
Number of study participants [N]	40	98	25	28	10	10	6	6	12	25
Female [N, %]	32 (77.5)	65 (65.3)	18 (72.0)	18 (64.3)	7 (70.0)	5 (50.0)	4 (75.0)	4 (75.0)	9 (75.0)	15 (60.0)
Age [median, range]	31 (19–55)	29 (21–57)	30 (19–55)	29 (21–57)	29 (21–55)	29 (23–55)	33 (24–55)	34 (22–55)	32 (27–42)	30 (23–37)
Disease duration^a^ [median, range]	1.5 (0–11)	na	2.0 (0–11)	na	2.0 (0–2)	na	0.0 (0–3)	na	1.0 (0–5)	na
Active disease^b^ [N, %]	24 (60.0)	na	13 (52.0)	na	4 (40.0)	na	4 (75.0)	na	9 (75.0)	na
EDSS score^c^ [median, range]	2.0 (1.0-3.5)	na	2.0 (1.0–3.0)	na	2.0 (1.0-2.5)	na	2.0 (1.0-3.5)	na	1.0 (1.0–3.0)	na

*MS*  Multiple sclerosis, *HD*  Healthy donor

^a^years from disease onset to blood sampling, ^b^Disease activity at time of blood sampling (acute = acute relapse, active disease stage; remission =clinical remission, inactive disease stage); ^c^Expanded Disability Status Scale

## Methods

**Fig. 1:**
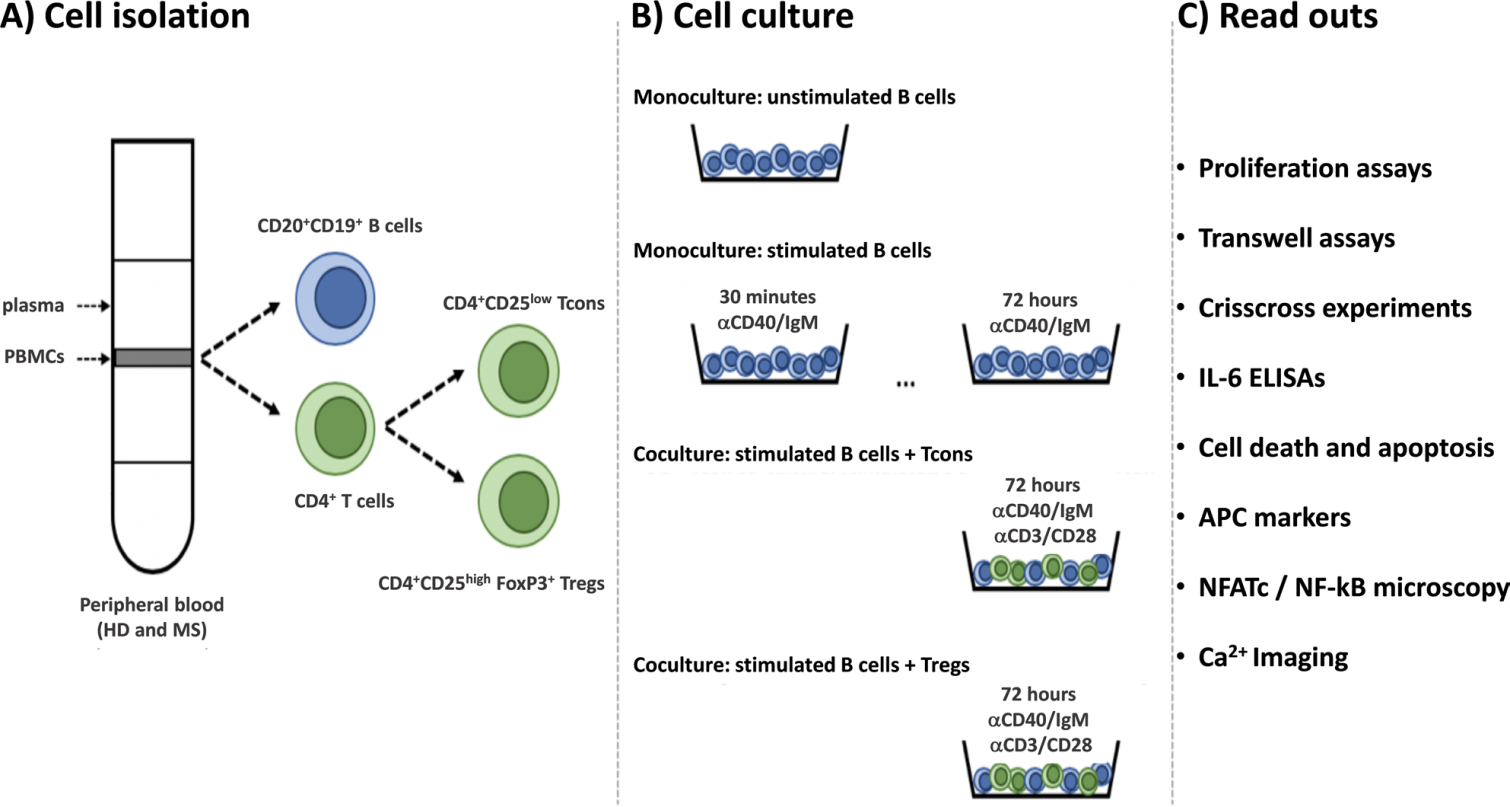
Workflow illustrating cell isolation, stimulation, culture conditions and downstream applications.** A** B cells, Tcons and Tregs were isolated from whole blood drawn from healthy donors and MS patients using commercially available kits. **B** Isolated B cells were stimulated with anti-IgM/anti-CD40. T cells were stimulated with a CD3/CD28 T cell activator. B cells were either cultured as monoculture, or in coculture with Tcons or Tregs at a 1:1 ratio. Unstimulated B cells were always cultured as negative control. **C** Cells were harvested at different timepoints to perform downstream assays, which included proliferation assays, IL-6 ELISAs, cell death and apoptosis assays, NFATc1 / NF-kB immunocytochemistry and microscopy, and live-cell calcium imaging

The experimental workflow (shown in Fig. 1) comprises cell separation from peripheral blood (including purity check by flow cytometry) and cell culture experiments with downstream applications (proliferation assays, crisscross experiments, transwell experiments, IL-6 secretion (by ELISA), determination of cell death and apoptosis (by flow cytometry), measurement of APC marker expression levels (HLA-DR, CD80 and CD86 by flow cytometry), single-cell Ca²^+^ imaging and NFATc1/NF-kB microscopy).

### Flow cytometry

A panel of fluorescence dye-labeled mAbs was used in different mixes to identify and characterize human B cells and T cells. All mAbs used for flow cytometry analyses are listed in Supplementary Table 1. Supplementary Fig. 3 shows gating hierarchies, including those for Ki-67 stainings (lymphocytes -> singlets -> B cells (CD19^+^/CD20^+^) and T cells (CD4^+^) -> live cells (where available) -> Ki-67. Flow cytometric data were acquired on a FACSCanto II flow cytometer, using CellQuest™ software (BD Biosciences), and analyzed using FlowJo™ (Version 10.7, Ashland, OR, USA).

### Cell isolation and purity assessment

#### Cell isolation

Peripheral blood mononuclear cells (PBMCs) were isolated from 40 to 80 ml whole blood by density gradient centrifugation (Bio & Sell, Feucht, Germany). Total CD19^+^ B cells and CD4^+^ T cells were negatively isolated from PBMCs using the Dynabeads^®^ Untouched Human B cells kit and the Dynabeads Untouched Human CD4 T cells kit, respectively, according to the manufacturer’s instructions (Thermo Fisher Scientific, Schwerte, Germany). Tregs were enriched from the total CD4^+^ T cell population using the Dynabeads^®^ Regulatory CD4+ CD25+ cells kit, according to the manufacturer’s protocol (Thermo Fisher Scientific). For subsequent transcriptome analysis, cultured B cells were either purified by positive isolation using CD19 MicroBeads (Miltenyi Biotec) (monoculture) or, following coculture with Tregs or Tcons, CD4^+^ cells were first depleted using CD4 MicroBeads (Miltenyi Biotec, Bergisch Gladbach, Germany) and B cells were then purified with CD19 MicroBeads.

#### Purity assessment

Following separation from peripheral blood samples, 2 × 10^5^ PBMCs, as well as 1 × 10^5^ purified B cells, Tregs and Tcons, were stained for pan-B cell surface marker CD20 to identify CD20^+^ total B cells, and/or for CD4, CD25 and FoxP3 allowing identification of CD4^+^CD25^high^FoxP3^+^ Tregs and CD4^+^CD25^low^FoxP3^−^ Tcons and analyzed by multi-color flow cytometry (Supplementary Figs. 1 and 2). Purity assessment revealed highly pure B cells, Tregs and Tcons (Supplementary Table 2A). Following immunomagnetic isolation from cell culture, total B cells were checked for purity by double staining for CD19 and CD4 to rule out T cell contamination, constantly revealing highly pure B cell preparations (Supplementary Fig. 2, Supplementary Table 2B).

#### Coculture and proliferation assays

Freshly isolated B cells were cultured either as monocultures or in cocultures with Tregs in different ratios. In some experiments Tcons were used as non-Treg controls. All cells were cultured in RPMI medium (Bio & Sell, Feucht, Germany) supplemented with 10% fetal bovine serum (FBS), 100 U/ml penicillin, and 100 µg/ml streptomycin at a concentration of 1 × 10^6^ cells/ml in a 96-well round-bottom plate containing 100 µl per well. B cells were stimulated with anti-IgM antibodies (12.5 µg/ml; Jackson ImmunoResearch, Ely, United Kingdom) and anti-CD40 antibodies (5 µg/ml; Miltenyi Biotec), while Tregs and Tcons were activated using a CD3/CD28 T cell activator (25 µl/ml; Stemcell Technologies, Cologne, Germany). Cells were maintained at 37 °C and 5% CO_2_ for up to 72 h, following which 2 × 10^5^ cells per condition and supernatants were harvested and used for subsequent experiments. Unstimulated B cells in monoculture served as control condition and were kept for 72 h before harvesting.

#### Proliferation assays

B cell proliferation was determined by flow cytometric assessment of intracellular expression of Ki-67, a mitosis marker which is closely related to cell proliferation [48–50]. Therefore, cultured cells were harvested and first surface-stained for CD19 and CD4 to distinguish between B cells and CD4^+^ T cells (Tregs or Tcons) and then intracellularly for Ki-67, identifying CD4^−^CD20^+^Ki-67^+^ proliferating B cells (Supplementary Fig. 3E). Successful activation of CD4^+^ T cells was confirmed by Ki-67 staining of Tcons following 48 h of cell culture in the presence of 25 µl/ml CD3/CD28 T cell activator (Supplementary Fig. 4).

#### Crisscross experiments

In addition to autologous B cell‒Treg cocultures carried out as proliferation assays (see above), B cells and Tregs obtained from 10 MS patients and 10 HD were tested as 10 experimental pairs in crisscross assays.

Crisscross design (exemplary for experimental pair #01):

- B cells obtained from MS patient #01 were cocultured with (i) autologous Tregs and (ii) Tregs obtained from HD #01, in a separate well.
- The same process was repeated for HD #01 B cells and MS #01 Tregs.
- This results in in four different conditions for each of the 10 experimental pairs: HD B cells/HD Tregs; HD B cells/MS Tregs; MS B cells/HD Tregs; MS B cells/MS Tregs.

#### Transwell experiments

To assess whether B cell‒Treg interaction was dependent upon direct cellular contact, conventional proliferation assays (outlined above) were paralleled by additional coculture experiments, which were performed in 24-well plates with transwell inserts (pore size 0.4 μm; Sarstedt, Nümbrecht, Germany), where B cells were cultured in the wells, with Tregs added to the transwell compartment.

### IL-6 ELISA

Quantities of 50 µl cell culture supernatant per well from conventional proliferation assays (outlined above) were harvested and stored at − 80 °C until measurement of the B cell-associated cytokine IL-6 using the Quantikine ELISA Kit (Bio-Techne GmbH (Wiesbaden, Germany). Cell culture medium was free of endotoxin as measured by ToxinSensor™ Gel Clot Endotoxin Assay Kit (GenScript Biotech, Rijswijk Netherlands).

### Dead and apoptotic cells

Cultured cells were harvested, surface stained for CD4 and CD19 (see above), followed by free amine staining with the LIVE/DEAD Fixable Red Dead Cell Stain Kit (Thermo Fisher Scientific) allowing quantification of CD4^−^CD19^+^ dead/necrotic B cells (Supplementary Fig. 3D). Early apoptotic B cells were identified by CD4/CD19 double staining and co-staining for Annexin V. B cells, treated for 3 h with 20 µM Staurosporine (Merck KGaA, Darmstadt, Germany) served as positive control (Supplementary Fig. 5).

### Expression of APC markers

To assess whether Tregs impact the antigen-presenting ability of B cells, expression levels of costimulatory receptors HLA-DR, CD80 and CD86 on B cells, either stimulated alone or in coculture with Tregs for 72 h, were determined by multi-color flow cytometry. CD4/CD19 double staining of harvested cells (see above) was completed by co-staining for HLA-DR, CD80 and CD86 and then MFIs (median fluorescence intensities) for each marker were determined in CD4^−^CD19^+^ B cells (Supplementary Fig. 3F).

### Single-cell Ca²^+^ imaging

Ca^2+^ signals in human B cells were assessed by single-cell live imaging using a custom recording chamber (Supplementary Fig. 6A), according to a previously established protocol [42]. Briefly, unstimulated B cells in monoculture or in coculture with prestimulated Tregs were loaded with 3 µM Fura-2 AM (Sigma-Aldrich, Steinheim, Germany), a commonly used calcium indicator [51], and inserted into the recording chamber. Ca^2+^ imaging was performed in HBSS buffer (with Ca^2+^ and Mg^2+^ but no phenol red; Thermo Fisher Scientific) containing 1.26 mM CaCl_2_ using a Nikon Eclipse Ti inverted fluorescent microscope (Nikon Europe B.V., Amsterdam, Netherlands) equipped with a 20× multi-immersion objective. Cells were alternately illuminated at 340 nm and 387 nm every 10 s for a total of 30 min, and emission (510 nm) was recorded using a CCD camera (Hamamatsu, Herrsching, Germany). Data acquisition was controlled by NIS Elements software (Version 5.21.01, Nikon). Cells were perfused in HBSS^+^ and kept at 37 °C throughout the experiment, with the help of a heating system. Following single-cell live imaging, cells were immediately fixed with 4% PFA (AppliChem, Darmstadt, Germany) in the recording chamber, followed by staining for CD20, CD4 and CD25 to distinguish B cells and Tregs (Supplementary Fig. 6B; mAbs used are listed in Supplementary Table 1). Fluorescent images were acquired using standard filter sets (AHF Analysetechnik, Tübingen, Germany), and then overlaid with the last frame of the live-cell imaging experiment, to identify Ca^2+^ responses at single-cell level. Ca^2+^ responses were analyzed using the Python Environment Spyder (Version 5.3.3; https://www.spyder-ide.org/).

### NFATc1/NF-kB microscopy

For intracellular localization of transcription factors NFATc1 (nuclear factor of activated T cells, cytoplasmic 1) and NF-κB (nuclear factor ‘kappa-light-chain enhancer’ of activated B cells), cultured B cells were harvested and transferred to 24-well coverslips. Cells were fixed with 4% PFA, permeabilized with 0.5% Triton X-100, washed with PBS, and blocked with 1% BSA/PBS solution. Staining was performed with unlabeled mAbs specific for human NFATc1, CD4 and CD20 and secondary anti-mouse Cy3 antibodies (mAbs used are listed in Supplementary Table 1). Nuclei were stained with DAPI, and translocation was quantified by defining regions of interest either around or within the nucleus for measuring the sum pixel intensity (after background correction), and the ratio (R) subsequently calculated between cytoplasmic and nuclear regions. The value *R* = 1 indicates equal distribution between the nucleus and cytoplasm, whereas values above/below 1 indicate predominant nuclear/cytoplasmic localization, respectively. Images were taken on an inverted confocal microscope (Nikon AR1, Nikon), and quantification was performed using ImageJ software (NIH, Bethesda, Maryland).

### Statistics

Statistical analyses were performed using GraphPad Prism software (Version 10; GraphPad, San Diego, CA, USA). Data were tested for normality and homogeneity of variance using the Shapiro‒Wilk test and the Brown‒Forsythe test, respectively. Differences between two groups were assessed using independent t-tests or non-parametric Mann‒Whitney U-tests. Welch’s one-way analyses of variance (ANOVAs) were performed to compare the means between more than two independent, normally distributed groups. P-values for pairwise comparisons were adjusted using Tukey’s correction for post-hoc comparisons between all groups and Dunnett’s T3 correction for planned comparisons between preselected conditions. Non-parametric Kruskal‒Wallis tests with Dunn’s post-hoc corrections were performed to compare more than two conditions with non-normally distributed data. Two-way ANOVA with Tukey’s multiple comparison was used for comparing groups on two different categorical variables. P-values of less than 0.05 were considered statistically significant.

## Results

### B cell responses to stimulation with anti-IgM/anti-CD40 are not altered in MS

The responsiveness of total B cells derived from peripheral blood samples of MS patients and HD to stimulation with anti-IgM/anti-CD40 was evaluated by single-cell live Ca^2+^ imaging (to assess early activation signals) and by in vitro proliferation assays (to assess downstream effects).

#### Ca^2+^ influx in anti-IgM/anti-CD40-stimulated B cells

**Fig. 2:**
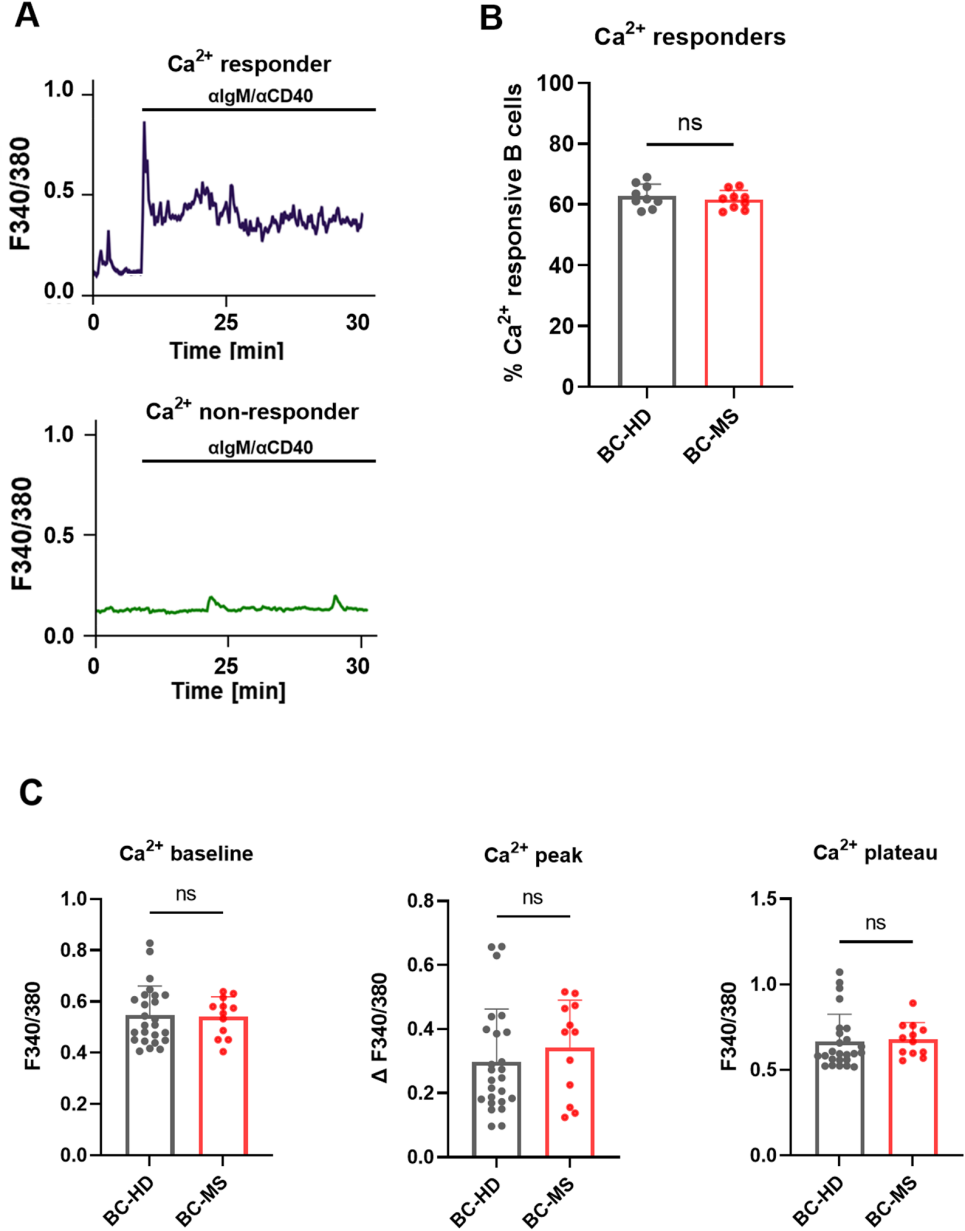
Ca^**2+**^ signaling patterns of human B cells in response to anti-IgM/anti-CD40 stimulation.** A** Single-cell Ca^2+^ imaging was performed as shown in Supplementary Figure S4. Following 5 min of baseline recordings, B cells were stimulated with anti-IgM/anti-CD40 (as indicated), and their Ca^2+^ responses were recorded over a period of 25 min. Responses were defined as Fura-2 ratio values exceeding the mean plus two standard deviations of the average Fura-2 ratios during 30 min of recordings from unstimulated B cells. Responsive B cells exhibited a Ca^2+^ peak immediately after stimulation and maintained elevated intracellular Ca^2+^ levels throughout the recording period. Non-responsive B cells showed no increase in intracellular Ca^2+^ following stimulation. **B** Analysis of Ca^2+^ influx in anti-IgM/anti-CD40-stimulated B cells from HD (*n* = 9) revealed that 62.9% were responsive, comparable with the 61.5% of responsive B cells observed in MS patients (*n* = 9) of the same age. **C** Direct comparisons also showed no differences between HD B cells (*n* = 25) and MS B cells (*n* = 13) in terms of baseline Ca^2+^ levels, Ca^2+^ peaks, and Ca^2+^ plateaus. Results are based on a minimum of three independent experiments, with more than 50 cells per experiment. Bar charts show means ± standard error of the mean (SEM); data points represent individual cells. Comparisons between groups were performed using Welch’s t-tests. BC = B cells; ns = non-significant

Using single-cell live imaging, we analyzed Ca^2+^ responses in B cells stimulated with anti-IgM/anti-CD40, obtained from 12 MS patients (median age 32 years; range 27–42; 9 female) and 25 age- and sex-matched HD (median age 30 years, range 23–37; 15 female). A rapid and sustained intracellular Ca^2+^ influx, defined as Fura-2 ratio values exceeding the mean plus two standard deviations of unstimulated B cells over 30 min of recordings, was observed in comparable proportions of MS-derived B cells (mean 61.5%, range 57.5–66.2) and HD-derived B cells (mean 62.9%, range 57.7–69.0; *P* = 0.432; Fig. 2A, B). Direct comparisons between MS and HD B cells also revealed no significant differences in baseline Fura-2 ratios (prestimulation), peak (in response to stimulation [ΔFura-2_340/380_]), or plateau Ca^2+^ influx (mean Fura-2_340/380_ ratios during the final 10 min of recording [Fura-2_340/380_]; Fig. 2C). Total B cells were additionally stained for CD27, a marker distinguishing memory B cells [52]. Interestingly, a trend towards higher peak and plateau Fura-2 values in CD27^−^ naïve B cells than in CD27^+^ memory B cells was observed (Supplementary Fig. 7).

#### Activation of NFATc1 and NF-ĸB in anti-IgM/anti-CD40-stimulated B cells

**Fig. 3:**
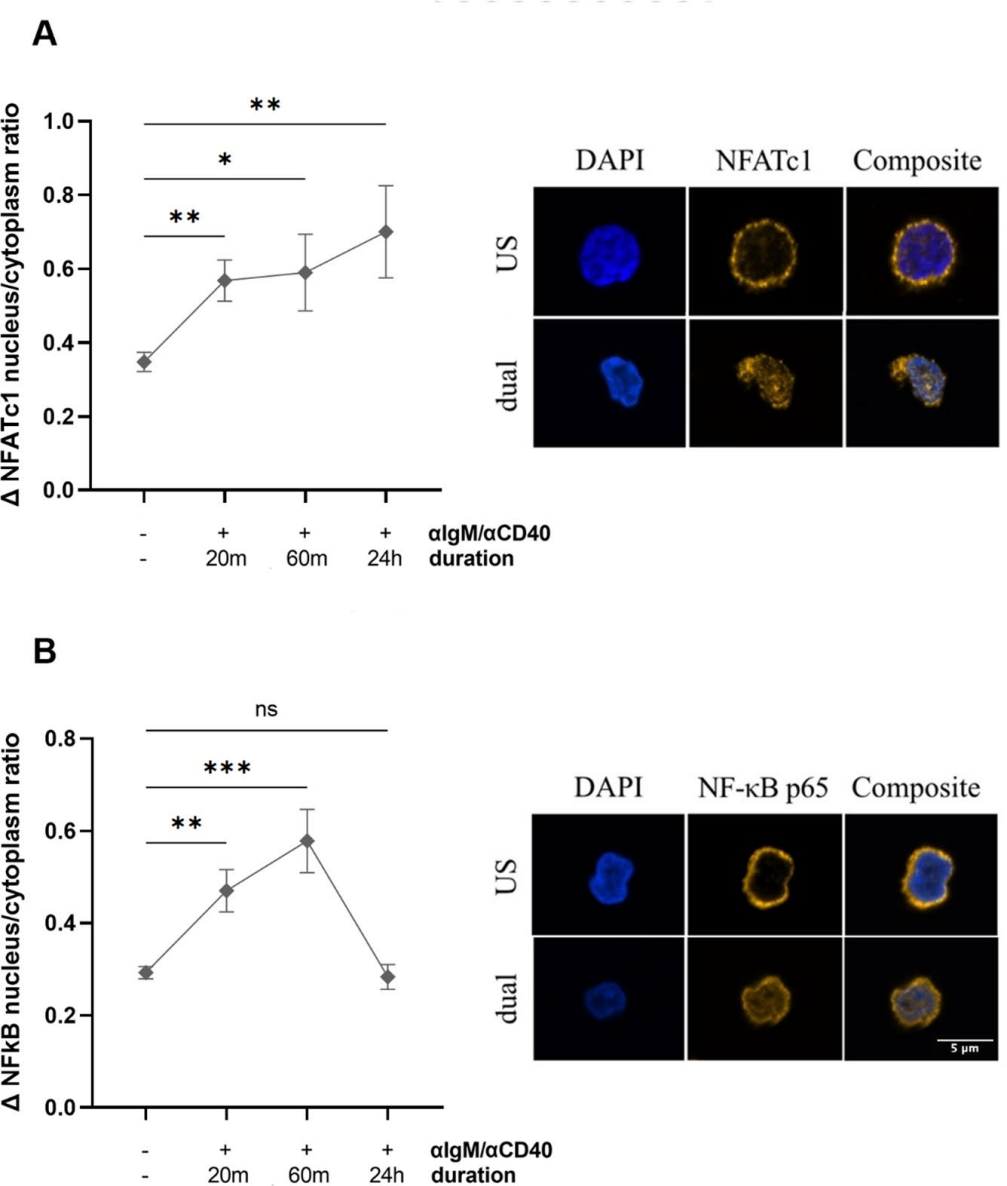
Time course of NFATc1 and NF-ĸB activation in anti-IgM/anti-CD40 stimulated B cells. Stimulation with anti-IgM/anti-CD40 induced significant nuclear translocation of NFATc1 (**A**, left panel) and NF-ĸB (**B**, left panel) in total B cells from HD (n = 18) after 20 and 60 min. After 24 h, NFATc1 activation was further increased (**A**, left panel), whereas NF-ĸB had been transported back to the cytoplasm (B, left panel). Representative immunofluorescence images of NFATc1 (**A**, right panel) and NF-ĸB (**B**, right panel) translocation are shown after 60 min, respectively. Data are presented as mean ± SEM. Comparisons were made using two-way ANOVA with Tukey’s test for multiple comparisons. ns = non-significant; US = non-stimulated; dual = anti-IgM/anti-CD40 stimulation. *P < 0.05; **P < 0.01; ***P < 0.001

Stimulation with anti-IgM/anti-CD40 triggers activation and nuclear translocation of NFATc1 and NF-ĸB, two transcription factors essential for B cell immune responses such as proliferation and survival. To investigate the early downstream effect of Ca^2+^ influx, we assessed the translocation rates for NFATc1 and NF-ĸB in total B cells isolated from 18 HD. Compared with non-stimulated B cells (mean ΔNFATc1 nucleus/cytoplasm ratio 0.35, range 0.17–0.57), anti-IgM/anti-CD40-stimulation induced a significant increase in NFATc1 translocation from the cytoplasm into the nucleus starting within 20 min and persisting up to 24 h in the majority of B cells (20 min: mean ΔNFATc1 nucleus/cytoplasm ratio 0.57, range 0.25–0.95, *P* = 0.001; 60 min: mean 0.59, range 0.28–1.59, *P* = 0.012; 24 h: mean 0.70, range 0.21–2.27, *P* = 0.006; Fig. 3A). NF-ĸB showed a similar early translocation pattern following stimulation (20 min: mean NF-ĸB nucleus/cytoplasm ratio 0.47, range 0.24–0.85, *P* < 0.001; 60 min: mean 0.58, range 0.32–0.86, *P* < 0.0001), but unlike NFATc1, its nuclear presence declined at 24 h: mean 0.28, range 0.13–0.40, *P* = 0.737), reaching levels comparable with non-stimulated B cells (mean ΔNF-ĸB nucleus/cytoplasm ratio 0.29, range 0.19–0.41; Fig. 3B).

#### Proliferative immune responses of anti-IgM/anti-CD40-stimulated B cells

**Fig. 4:**
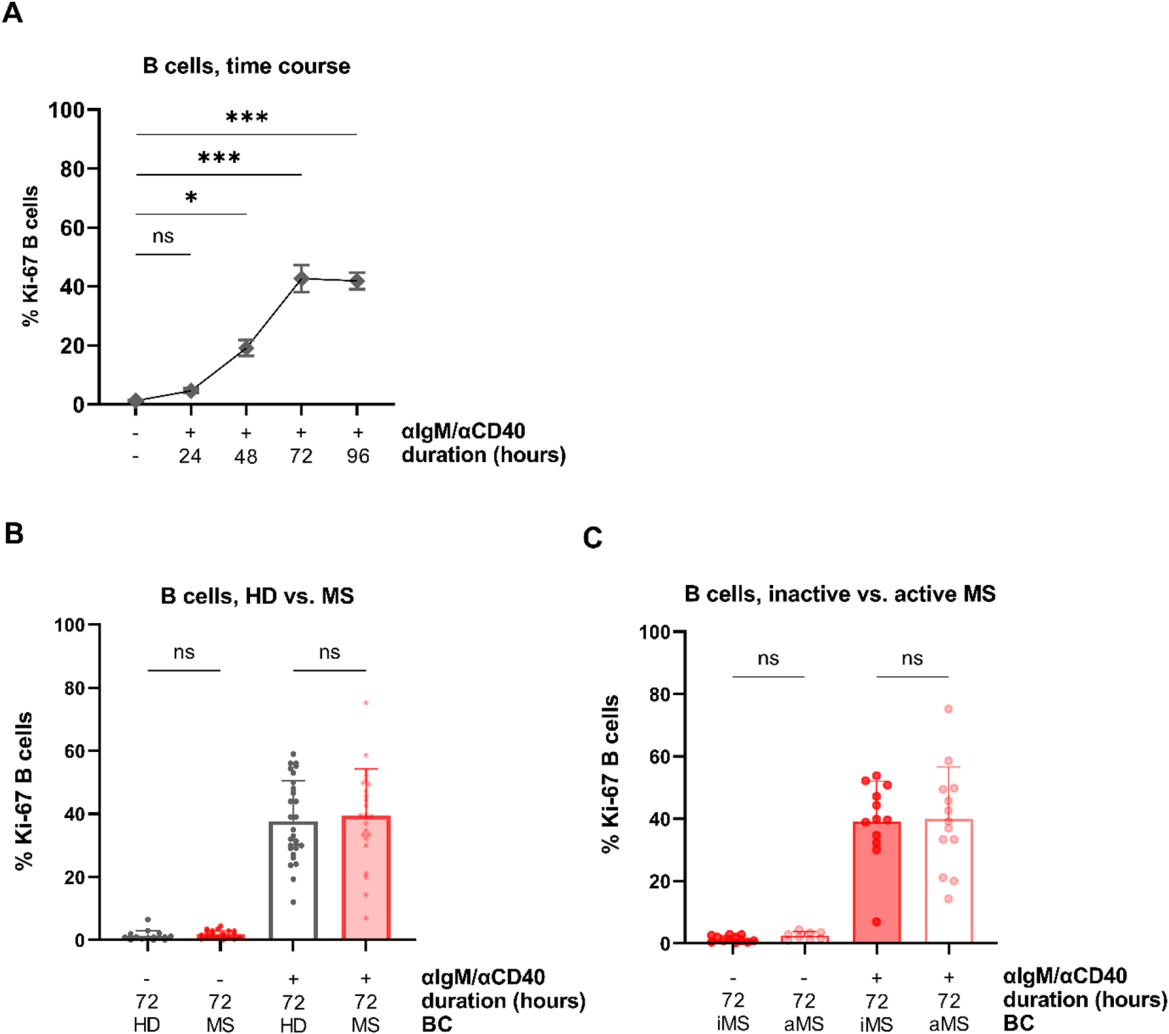
Proliferative immune responses of anti-IgM/anti-CD40-stimulated B cells in vitro. **A** Stimulation with anti-IgM/anti-CD40 significantly increased the percentage of proliferating B cells in a time-dependent manner, as assessed by intracellular staining for the mitosis marker Ki-67. B cells from 5 HD were stimulated and cultured for up to 96 h. Compared with unstimulated controls, the proportion of Ki-67^+^ proliferating B cells was significantly higher after 72 and 96 h of stimulation. **B** After 72 h of stimulation, the proliferative response of total B cells from patients with MS (n = 25) and HD (n = 28) did not differ. **C** No significant differences were observed between patients with inactive MS (iMS, n = 12) and patients with active MS (aMS, n = 13). The bar charts show means, error bars representing standard deviation, and individual data points. Comparisons between two groups were performed using Welch’s t-tests; comparisons among more than two groups were conducted using one-way ANOVAs followed by Kruskal‒Wallis tests with Dunn’s post-hoc corrections; BC = B cells; ns = non-significant. *P < 0.05; **P < 0.01; ***P < 0.001

To assess downstream proliferative responses following BCR and CD40 costimulation, in vitro proliferation assays were performed on B cells stimulated with anti-IgM/anti-CD40. A total of 2 × 10^5^ B cells isolated from peripheral blood samples of five HD were stimulated in monocultures for 24, 48, 72, and 96 h. Intracellular expression of Ki-67, a widely used cell proliferation marker [48–50], was quantified by flow cytometry. The percentage of Ki-67^+^ B cells increased over time, peaking at 72 h (mean 42.7%, range 31.5–58.6) and remaining elevated at 96 h (mean 41.9%, range 36.8–51.0; Fig. 4A). Based on these results, all subsequent cell culture experiments were conducted with a 72-hour stimulation period.

Next, B cells obtained from peripheral blood of 25 MS patients (median age 30 years; range 21–55; 18 female) and 28 age- and sex-matched HD (median age 30 years, range 23–56; 18 female) were evaluated for proliferative responses to anti-IgM/anti-CD40 stimulation. In line with the Ca^2+^ imaging results, no significant differences in Ki-67 expression were observed between MS and HD B cells, either at baseline (MS: mean 1.8%, range 0.2–4.4; HD: mean 1.3%; range 0.0–6.5; *P* > 0.999) or after 72 h of stimulation (MS: mean 39.6%, range 6.9–75.2; HD: mean 37.7%, range 12.0–59.0; *P* > 0.999; Fig. 4B). Furthermore, proliferative responses after 72 h were similar between patients with inactive disease (*n* = 12; mean, 39.2%; range, 6.3–53.8) and those with active disease (*n* = 13; mean, 40.0%; range, 14.3–75.2; *P* > 0.999; Fig. 4C), indicating that total B cells from MS patients retain a normal proliferative capacity in response to BCR/CD40 stimulation, irrespective of disease activity status.

### The Treg-mediated suppression of anti-IgM/anti-CD40-induced B cell proliferation is Ca^2+^ independent and reduced in MS

#### The Treg-mediated B cell suppression in vitro is dose-dependent

**Fig. 5:**
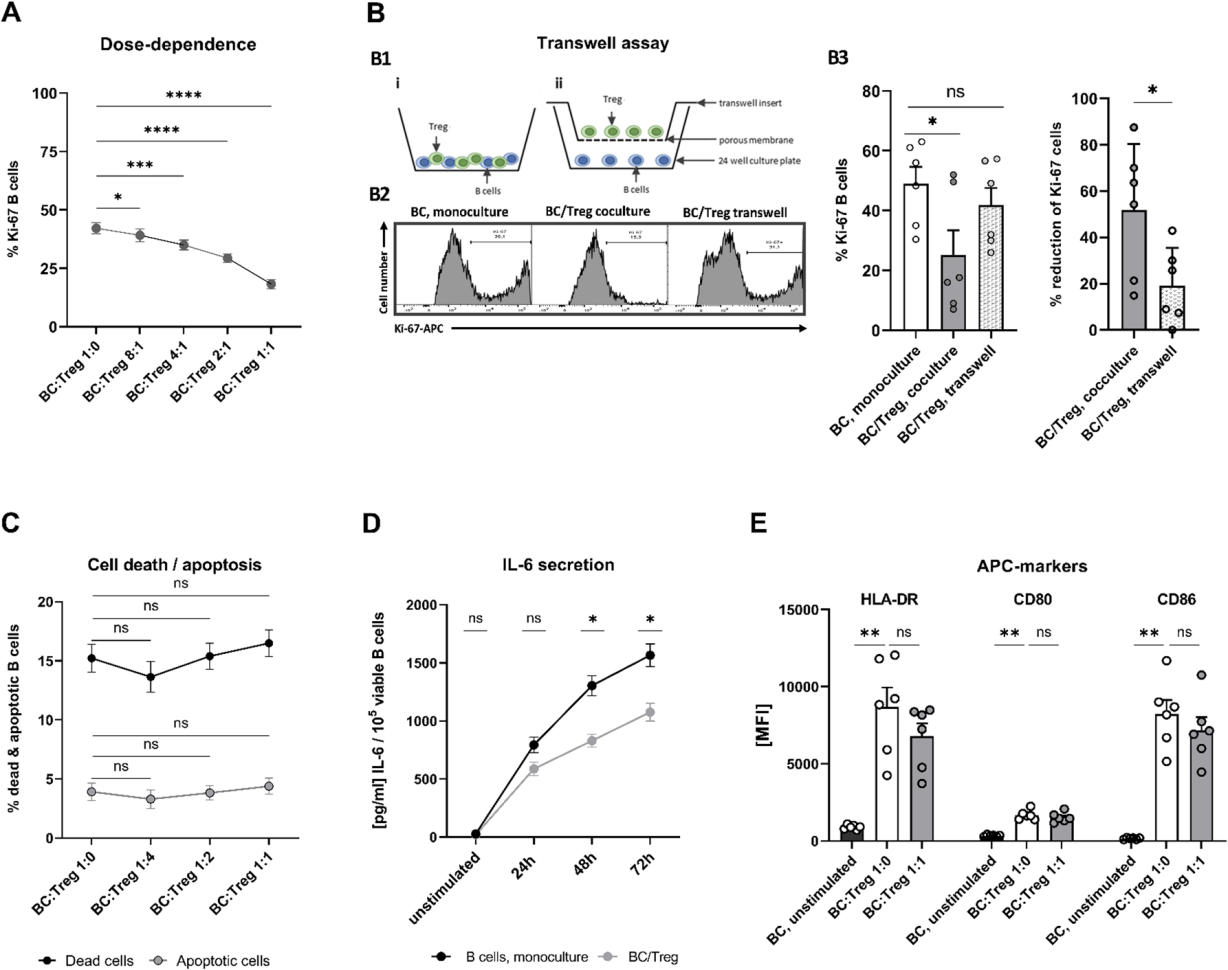
Characteristics of Treg-mediated B cell suppression in vitro. **A** dose dependence of Treg-mediated B cell suppression, assessed by in vitro proliferation assays. A total of 2 × 10^5^ purified B cells from HD (*n* = 3) were stimulated with anti-IgM/anti-CD40 for 72 h and cocultured with increasing Treg numbers (from left to right). Suppression of B cells increased with higher Treg numbers, with the greatest reduction of Ki-67^+^ B cells observed at a 1:1 B cell: Treg ratio. **B** Transwell assays: (B1) Cell culture conditions for the assessment of cell contact dependency of Treg suppressive effects on B cell proliferation: (i) In standard coculture conditions, B cells stimulated with anti-IgM/anti-CD40 were cultured alone or with anti-CD3/CD28-stimulated Tregs (1:1 ratio). 4 × 10^5^ cells/well were seeded in 24 well flat bottom cell culture plates. (ii) In transwell conditions, 2 × 10^5^ B cells in 800 µL stimulation medium were cultured per well. 2 × 10^5^ Tregs in 200 µL stimulation medium were cultured in a transwell insert with a pore size of 0.4 μm, to prevent direct cellular contact between B cells and T cells. (B2) Flow cytometry histogram of intracellular Ki-67 expression in B cells. (B3) A significantly greater reduction in Ki-67^+^ B cells was observed with B cells under direct coculture conditions, indicating that Treg-mediated suppression of B cell proliferation is at least partially cell contact-dependent. **C** Treg-mediated induction of apoptosis and cell death in B cells, as determined by coculture experiments with 2 × 10^5^ purified B cells stimulated with anti-IgM/anti-CD40 for 72 h and increasing numbers of autologous, CD3/CD28-stimulated, highly enriched Tregs. B cell apoptosis and death were determined by surface staining for CD19 and either intracellular staining with Annexin V (Supplementary Figure S5) or by free amine staining using the LIVE/DEAD Fixable Red Dead Cell Stain Kit (Supplementary Figure S3D). Both, the percentages of necrotic (HD, *n* = 6) and apoptotic B cells (HD, *n* = 6) did not significantly increase with increasing Treg numbers (from left to right). **D** Treg-mediated suppression of IL-6 production by B cells. Purified B cells from HD (*n* = 6; 2 × 10^5^ cells) were stimulated with anti-IgM/anti-CD40 for 24, 48, and 72 h either alone or in coculture with autologous, CD3/CD28-stimulated, highly enriched Tregs at a 1:1 ratio (“B cell: Treg”). IL-6 concentrations were measured using Quantikine ELISA Kits, resulting in significantly reduced IL-6 levels in the presence of Tregs after 48 and 72 h. **E** Expression levels of APC markers on B cells. Purified B cells from HD (*n* = 6; 2 × 10^5^ cells) were stimulated with anti-IgM/anti-CD40 for 72 h either alone or in coculture with autologous, CD3/CD28-stimulated, highly enriched Tregs at a 1:1 ratio and surface expression levels of HLA-DR, CD80 and CD86 were determined by multicolor flow cytometry (Supplementary Fig. 3), revealing (i) a strong increase for all APC markers tested following anti-IgM/anti-CD40 stimulation (ii) only slight and non-significant downregulation of these markers by coculture with Tregs. Percentages of proliferating B cells in panels (**A**) and (**B**) were determined by intracellular Ki-67 staining, along with surface markers CD19 (B cells) and CD4 (T cells) (Supplementary Figure S3E). Data in (**A**), (**C**), (**D**) and (**E**) are presented as mean ± SEM. In (D) IL-6 concentrations are expressed as [pg/ml] per 10^5^ cells viable B cells. The bar charts in (B3) show means, error bars representing SEM and individual data points of percentages of Ki-67^+^ proliferating B cells (left hand side) and of corresponding Treg-mediated reduction of Ki-67^+^ cells (% reduction = [(Ki-67% in monoculture – Ki-67% in coculture) / Ki-67% in monoculture] × 100; right hand side; 100% reduction defined as complete suppression of the proliferative response induced by anti-IgM/anti-CD40 compared with the corresponding B cell monoculture from the same donor). Comparisons between two groups were performed using Welch’s t-tests. Comparisons across multiple conditions were analyzed by two-way ANOVA with Tukey’s multiple comparisons test. BC = B cells; ns = non-significant. *P<0.05; **P<0.01; ***P<0.001; **P<0.0001

To determine whether the suppressive effect of Tregs on proliferation is influenced by the B cell: Treg ratio, in vitro proliferation assays were conducted using 2 × 10^5^ purified B cells obtained from three HD, which were stimulated with anti-IgM/anti-CD40 and cocultured for 72 h with increasing numbers of autologous, CD3/CD28-stimulated, highly enriched Tregs. A clear dose-dependent suppression of B cell proliferation was observed, as measured by intracellular Ki-67 expression. The most pronounced reduction in the proportion of Ki-67^+^ B cells occurred at a 1:1 ratio of B cells to Tregs (Fig. 5A). Based on these findings, a 1:1 B cell: Treg ratio was used for all subsequent experiments.

#### Cell–cell contact is necessary for Treg-mediated B cell suppression in vitro

To determine whether the suppressive effect of Tregs on anti-IgM/anti-CD40-induced B cell proliferation required direct cellular contact, B cells and Tregs were isolated from six HD and a total of 2 × 10^5^ B cells was stimulated with anti-IgM/anti-CD40 for 72 h alone or in coculture with autologous, CD3/CD28-activated, highly enriched Tregs at a 1:1 ratio. In parallel to standard coculture conditions, a transwell setup was used to physically separate Tregs and B cells while allowing soluble factors to diffuse, thereby preventing direct cellular contact. Proliferation of B cells was assessed via flow cytometry by staining for CD19 (B cell surface marker), CD4 (T cell surface marker), and the intracellular proliferation marker Ki-67. As shown in Fig. 5B, direct coculture with Tregs resulted in a significantly stronger reduction in the proportion of Ki-67^+^ B cells (mean 51.9%, range 14.9–87.5) than in the transwell condition (mean 19.2%, range 0.8–42.9, *P* = 0.034). These results indicate that Treg-mediated suppression of B cell proliferation is at least partially dependent on direct cellular contact.

#### Tregs do not induce significant cell death and apoptosis in B cells in vitro

To evaluate whether Treg-mediated suppression of B cells involves the induction of apoptosis and cell death, B cells and Tregs were isolated from 12 HD and a total of 2 × 10^5^ B cells was stimulated with anti-IgM/anti-CD40 for 72 h alone or in coculture with increasing numbers of autologous, CD3/CD28-stimulated, highly enriched Tregs. Apoptotic and dead B cells were identified by multicolor flow cytometry using either or a fixable viability dye (necrosis, Supplementary Fig. 3) or Annexin V (apoptosis, Supplementary Fig. 5). Both, the percentages of necrotic (*n* = 6; 1:1 B cell: Treg ratio: mean 16.5%, range 12.5–19.0; 1:0 B cell: Treg ratio: mean 15.2%, range 11.1–18.5; *P* = 0.459; Fig. 5C) and apoptotic B cells (*n* = 6; 1:1 B cell: Treg ratio: mean 4.4%, range 2.0–6.9); 1:0 B cell: Treg ratio: (mean 3.9%, range 1.8–5.8; *P* = 0.655; Fig. 5C) did not significantly increase with increasing Treg numbers. These findings suggest that, under the conditions tested, Treg-mediated suppression of B cell responses is primarily non-cytotoxic in nature.

#### IL-6 production is reduced in B cells when cocultured with Tregs

To determine whether Treg-mediated B cell suppression affects cytokine production by B cells, in vitro proliferation assays were carried out with B cells and Tregs obtained from six HD. Supernatants were harvested at 24, 48, and 72 h and analyzed for IL-6 levels by ELISA. Compared with B cells stimulated with anti-IgM/anti-CD40 in monoculture, coculture with CD3/CD28-activated Tregs (1:1 ratio) resulted in a significant reduction in IL-6 levels at 48 and 72 h (Fig. 5D). Normalization of IL-6 secretion to viable B cell counts showed that Tregs reduced IL-6 production per 10^5^ B cells to a similar extent as the unnormalized concentrations, confirming that reduced cytokine levels are not solely a consequence of fewer proliferating cells. These results indicate that, beyond inhibiting proliferation, Tregs suppress pro-inflammatory cytokine secretion by B cells. These IL-6 experiments in HD (Fig. 5D) were primarily designed as a proof of principle to demonstrate that Treg-mediated suppression extends from B cell proliferation to IL-6 production and to establish the experimental conditions used for subsequent MS vs. HD comparisons.

#### Expression levels of APC markers on B cells increase following CD40/IgM stimulation and are not significantly affected by coculture with Tregs

To evaluate whether Treg downregulate B cell antigen-presentation capacities in vitro, B cells and Tregs were isolated from six HD and a total of 2 × 10^5^ B cells was stimulated with anti-IgM/anti-CD40 for 72 h alone or in coculture with increasing numbers of autologous, CD3/CD28-stimulated, highly enriched Tregs. After 72 h of cell culture, cells were harvested and the surface expression levels of HLA-DR, CD80, and CD86 on B cells were determined by multicolor flow cytometry (Supplementary Fig. 3F). Compared with non-stimulated B cells (HLA-DR, mean 901, range 707–1001.3 [median fluorescence intensity, MFI]; CD80, mean 339, range 281–424 and CD86, mean 161, range 99–220 [MFI]), we found a significant increase in MFIs for all APC markers on anti-IgM/anti-CD40 stimulated B cells (HLA-DR, mean 8683, range 4256–12046 [MFI], *P* = 0.005; CD80, mean 1683, range 1392–2246 [MFI], *P* = 0.005 and CD86, mean 8241, range 5158–11684 [MFI], *P* = 0.002). This CD40/IgM induced expression of APC markers on B cells was only slightly and non-significantly lowered by 1:1 coculture with Tregs (HLA-DR, mean 6794, range 3729–8468 [MFI], *P* = 0.180; CD80, mean 1531, range 1164–2072 [MFI], *P* = 0.485 and CD86, mean 7172, range 4472–10760 [MFI], *P* = 0.394. Figure 5E). These data indicate that the observed decrease in Ki-67 expression reflects suppression of cell-cycle entry rather than a gross down-regulation of classical APC surface markers.

#### Treg-mediated suppression of B cell proliferation is reduced in MS

**Fig. 6:**
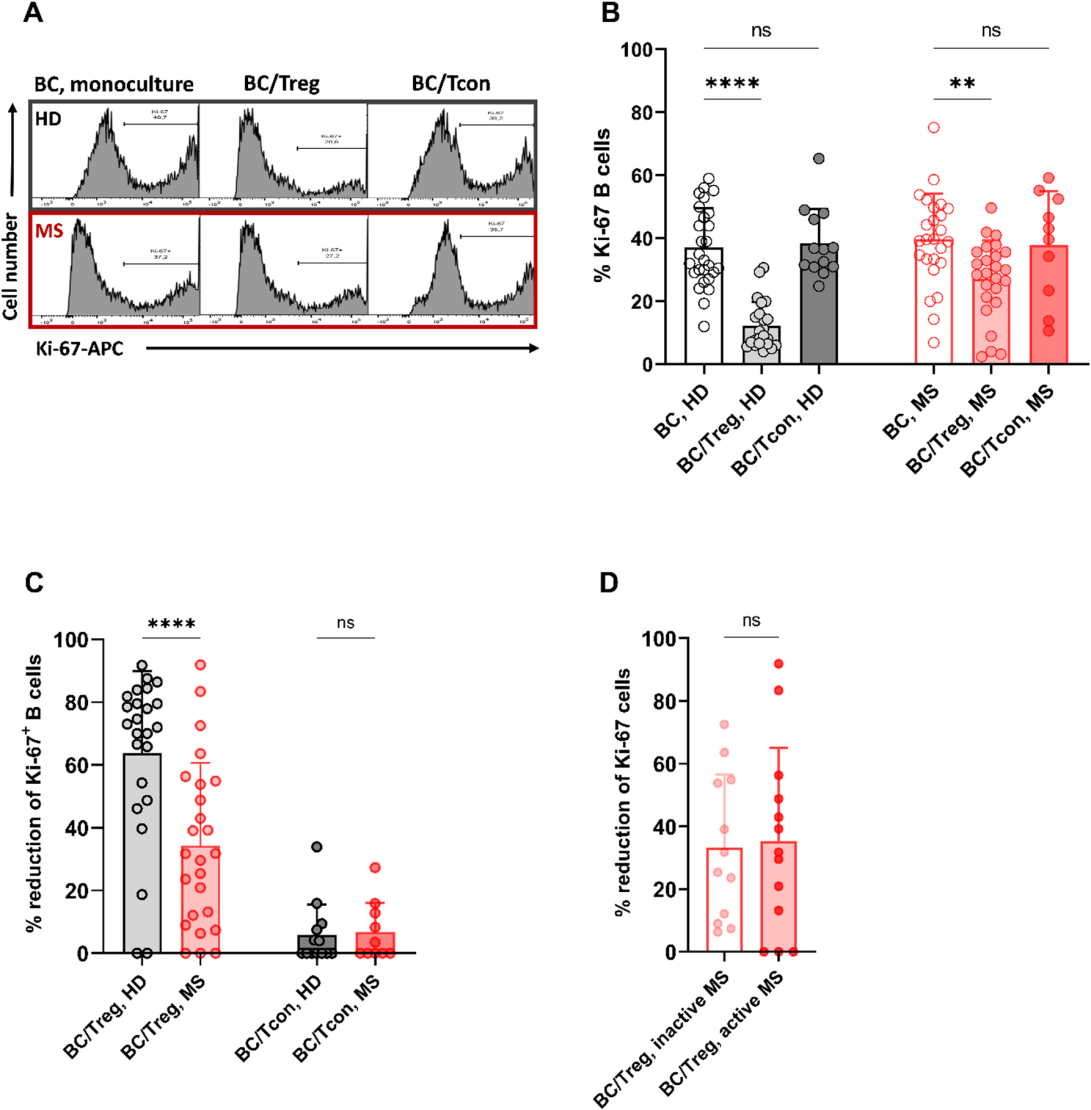
Treg-mediated suppression of anti-IgM/anti-CD40-induced B cell proliferation in MS-derived cells. In vitro proliferation assays were performed with 2 × 10^5^ purified B cells from 25 MS patients and 28 age- and sex-matched HD. B cells were stimulated with anti-IgM/anti-CD40 for 72 h, either alone (monoculture) or in coculture with highly enriched CD3/CD28-stimulated, autologous Tregs or Tcons (1:1 ratio). **A** Flow cytometry analysis of intracellular Ki-67 expression in B cells. Histograms show lower percentages of Ki-67^+^ B cells when cocultured with Tregs but not with Tcons. Treg-mediated reduction of Ki-67^+^ B cells was stronger in HD than in MS. **B** Both MS- and HD-derived Tregs but not MS- (*n* = 10) and HD-derived (*n* = 13) Tcons significantly reduced the percentages of proliferating Ki-67^+^ B cells. **C** However, MS-derived Tregs showed significantly reduced suppressive capacity compared with HD-derived Tregs, whereas the inhibitory effect observable in cocultures containing Tcons was comparably low in MS and HD. **D** Treg-mediated suppression of B cell proliferation did not differ between patients with inactive MS (*n* = 13) and patients with active MS (*n* = 12). In (B-D) bar charts show means, error bars representing SEM and individual data points. (% reduction = [(Ki-67% in monoculture – Ki-67% in coculture) / Ki-67% in monoculture] × 100; 100% reduction defined as complete suppression of the proliferative response induced by anti-IgM/anti-CD40 compared with the corresponding B cell monoculture from the same donor). Comparisons between two groups were performed using Welch’s t-tests. Comparisons among more than two groups were conducted using one-way ANOVA with Kruskal‒Wallis tests with Dunn’s post-hoc corrections. BC = B cells; ns = non-significant. *P < 0.05; **P < 0.01; ****P < 0.0001

Autologous B cells and Tregs or Tcons that served as non-Treg controls obtained from peripheral blood samples of 25 MS patients (median age 30 years; range 21–55; 18 female) and 28 age- and sex-matched HD (median age 30 years, range 23–56; 18 female) were analyzed in cell culture experiments. Proliferative responses were assessed by intracellular Ki-67 staining (Fig. 6A, Supplementary Fig. 3). In both MS- and HD-derived samples, the presence of Tregs significantly reduced B cell proliferation upon anti-IgM/anti-CD40 stimulation ([MS: Ki-67^+^ B cells in monoculture: mean 39.6%, range 6.9–75.2; coculture: mean 27.0%, range 2.4–49.6, *P* = 0.002; HD: monoculture: mean 37.1%, range 12.0–59.0; coculture with Tregs: mean 12.2%, range 4.0–30.6, *P* < 0.0001; Fig. 6B). No significant reduction in B cell proliferation was observed when cocultured with Tcons (MS: Ki-67^+^ B cells mean 37.8%, range 10.6–59.2, *P* = 0.777; HD: mean 38.3%, range 24.8–65.3, *P* = 0.745; Fig. 6B). However, when directly comparing the extent of Treg-mediated suppression, the reduction was significantly less pronounced in MS (mean suppression, as measured by % reduction of Ki-67^+^ B cells: mean 34.3%, range 0.0–91.9) versus HD (mean 63.8%, range 0.0–91.7, *P* = 0.0003; Fig. 6B), whereas the suppressive effect observable in cocultures containing Tcons was comparably low in both MS and HD (MS: Ki-67^+^ B cells mean 6.8%, range 0.0–9.1; HD: mean 5.8, range 0.0–8.2, *P* = 0.802; Fig. 5B). This reduced suppressive effect was observed irrespective of disease activity, as no significant difference was found between patients in remission (*n* = 12, mean suppression: 33.3%, range 6.3–72.5) and patients with active MS (*n* = 13, mean suppression: 35.2%, range 0.0–91.9, *P* = 0.857; Fig. 6C).

#### Treg-mediated inhibition of IL-6 production by B cells is decreased in MS

**Fig. 7:**
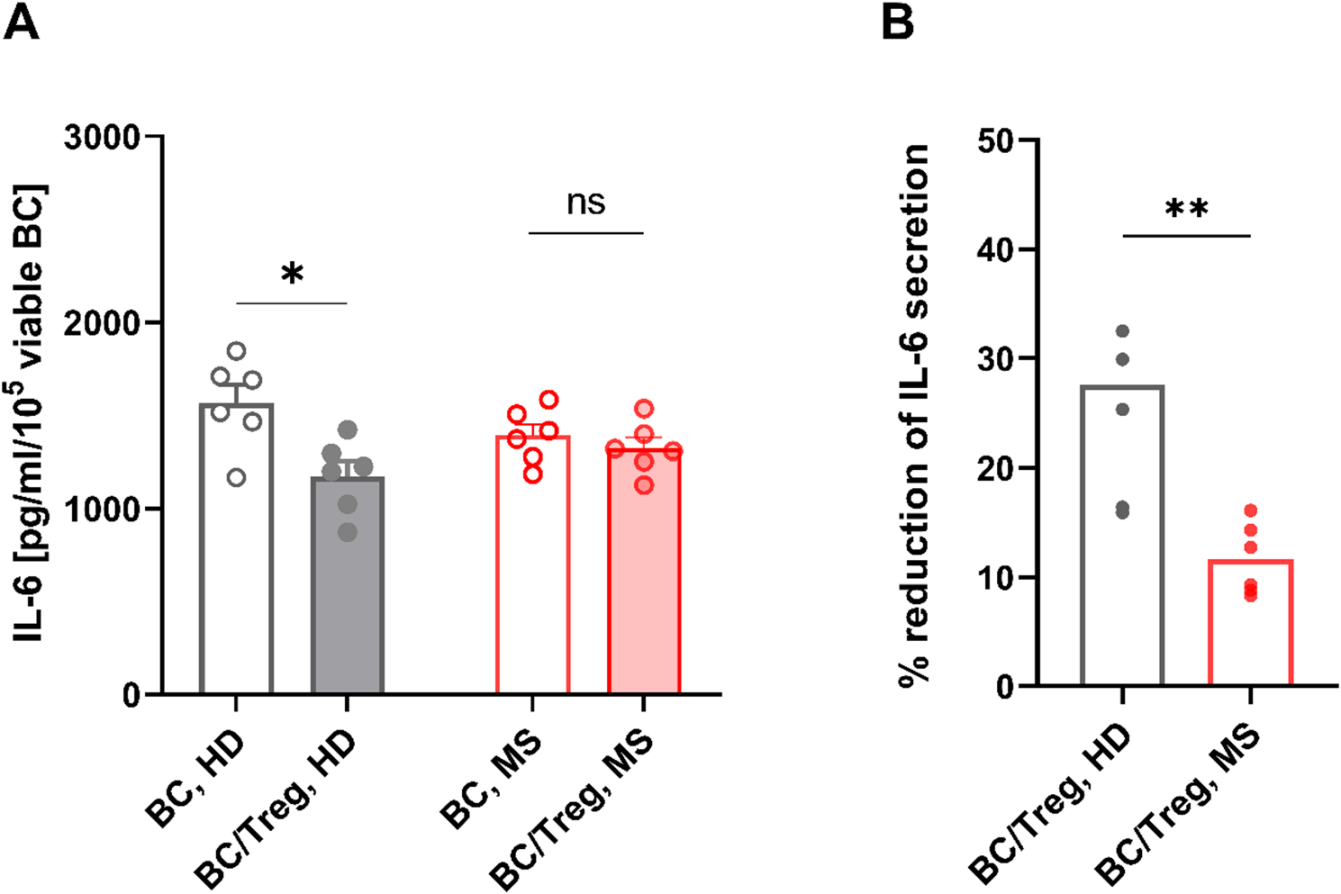
Treg-mediated suppression of IL-6 secretion by B cells in MS and HD. These MS vs. HD comparisons build on the proof-of-principle IL-6 data in HD shown in Fig. 5D. **A** IL-6 secretion by B cells was determined by Quantikine ELISA (Biotechne) in supernatants from cell culture experiments with 2 × 10^5^ purified B cells (MS, *n* = 6 (median age 33 years; range 24–55; 4 female); HD, *n* = 6 (median age 34 years, range 22–55; 4 female)) stimulated with anti-IgM/anti-CD40, either alone or in 1:1 coculture with autologous, CD3/CD28-stimulated, highly enriched Tregs for 72 h. **A** IL-6 secretion by stimulated B cells (normalized to viable cells) was significantly reduced by coculture with Tregs in HD but not in MS. **B** Treg-mediated suppression of IL-6 production was significantly decreased in MS-derived samples. Charts show means, error bars representing SEM and individual data points. Comparisons between two groups were performed using Welch’s t-tests. Comparisons among more than two groups were conducted using one-way ANOVA with Kruskal‒Wallis tests with Dunn’s post-hoc corrections. BC = B cells; ns = non-significant. *P < 0.05; **P < 0.01

Building on the proof-of-principle IL-6 experiments in HD (Fig. 5D), we next compared Treg-mediated inhibition of IL-6 production by B cells between MS patients and HD (Fig. 7). To this end, total B cells and Tregs, obtained from six MS patients (median age 33 years; range 24–55; 4 female) and six age- and sex-matched HD (median age 34 years, range 22–55; 4 female) were stimulated with anti-IgM/anti-CD40 either alone or in coculture with increasing numbers of autologous, CD3/CD28-stimulated, highly enriched Tregs. After 72 h, supernatants were harvested and tested for IL-6 using ELISA, revealing that in HD- but not in MS-derived samples, the presence of Tregs significantly reduced IL-6 secretion by stimulated B cells ([MS: B cells in monoculture: mean 1294.8, range 1341.5–1674.7 [pg/ml normalized to 10^5^ viable B cells]; coculture: mean 1322.7, range 1125.7–1535.7, *P* = 0.435; HD: monoculture: mean 1565.4, range 1167.8–1845.1 [pg/ml normalized to 10^5^ viable B cells]; coculture: mean 1172.6, range 872.3–1421.9, *P* = 0.011; Fig. 7A). Accordingly, the Treg-mediated suppression of IL-6 secretion by stimulated B cells was significantly lower in MS patients (% reduction of IL-6: mean 11.6%, range 8.3–16.1) than in HD (mean 25.0%, range 15.9–32.5, *P* = 0.044; Fig. 7B).

#### Crisscross experiments reveal reduced Treg suppression in MS arises from both Tregs and B cells

**Fig. 8:**
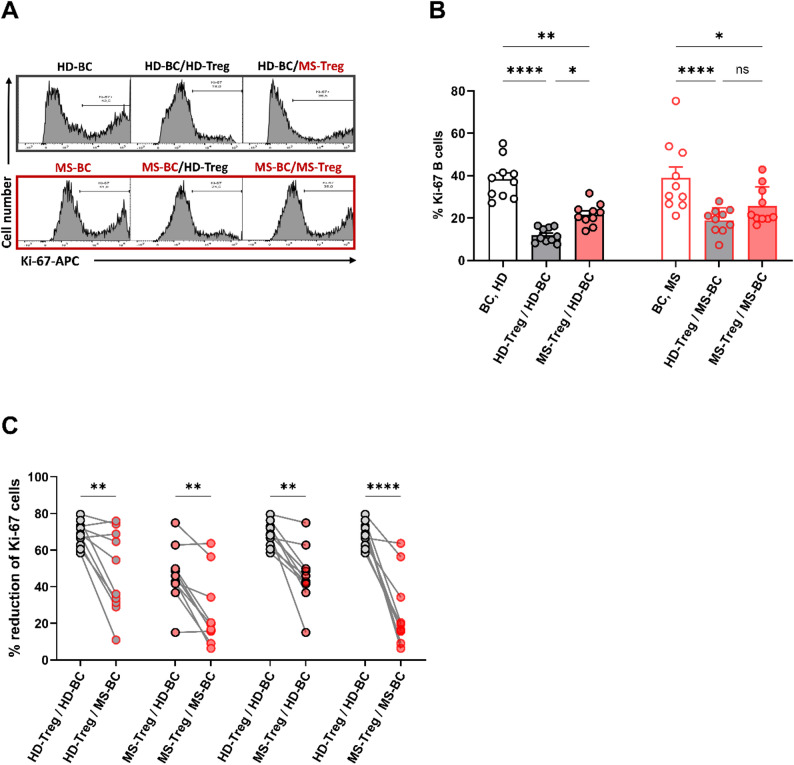
Treg-mediated reduction of B cell proliferation in crisscross cultures. Mixed coculture experiments were carried out with B cells and Tregs obtained from 10 patients with MS (*n* = 6; in inactive stage of disease, *n* = 4 in active stage of disease; 7 female) and 10 age- and sex-matched HD tested in paired design resulting in four conditions for each experimental pair (HD B cells/HD Treg, HD B cells/MS Treg, MS B cells/HD Treg, MS B cells/MS Treg) (see Methods section). HD B cells (2 × 10^5^ each) were stimulated for 72 h with anti-IgM/anti-CD40 alone or in the presence of CD3/28 activated autologous Tregs or Tregs derived from one MS patient (1:1 ratio) or vice versa and B cell proliferation was determined by intracellular Ki-67 staining. **A** Flow cytometry analysis of intracellular Ki-67 expression in B cells. Histograms show lower percentages of Ki-67^+^ B cells when cocultured with HD Tregs compared to MS Tregs. **B** Left side shows the percentages of HD-derived Ki-67^+^ B cells stimulated alone or in coculture with either autologous Tregs or Tregs derived from one MS patient (experimental pair); right side shows the percentages of MS-derived Ki-67^+^ B cells stimulated alone or in coculture with either autologous Tregs or Tregs derived from one HD (experimental pair). **C** Corresponding Treg-mediated reduction of Ki-67^+^ cells (% reduction = [(Ki-67% in monoculture – Ki-67% in coculture) / Ki-67% in monoculture] × 100; 100% reduction defined as complete suppression of the proliferative response induced by anti-IgM/anti-CD40 compared with the corresponding B cell monoculture from the same donor. Within-donor pairings show that (i) coculture with HD-derived Tregs resulted in a greater reduction in B cell proliferation, both for autologous and MS-derived B cells, than coculture with MS-derived Tregs and (ii) that MS-derived B cells exhibited reduced susceptibility to both autologous and HD-derived Tregs when compared with HD-derived B cells. Bar charts in (B) show means ± SEM with individual data points; data points and connecting lines in (C) represent paired samples. Comparisons are made using two-way ANOVA with Tukey’s multiple comparisons tests. BC = B cells; ns = non-significant; *P < 0.05; **P < 0.01. ****P < 0.0001

To determine whether the reduced Treg-mediated suppression of B cells seen in MS is due to impaired Treg function, reduced responsiveness of B cells to Treg signals, or both, we performed crisscross coculture experiments with Tregs and B cells, obtained from age- and sex-matched pairs of MS patients (median age 29, range 21–55; 7 female) and HD (median age 29 years, range 23–55; 5 female) (10 patients vs. 10 donors; see Table 1 and Supplementary Table 3). Cocultures were set up in paired design resulting in four conditions (HD B cells/HD Treg, HD B cells/MS Treg, MS B cells/HD Treg, MS B cells/MS Treg) for each experimental pair (see Methods section). As shown in Fig. 8, HD Tregs induced significantly greater suppression of B cell proliferation in both HD- and MS-derived B cells than MS Tregs. In addition, MS B cells exhibited significantly less susceptibility to Treg-mediated suppression than HD B cells, regardless of whether they were cocultured with HD- or MS-derived Tregs.

#### Tregs do not affect BCR/CD40-induced Ca^2+^ signaling in B cells

**Fig. 9:**
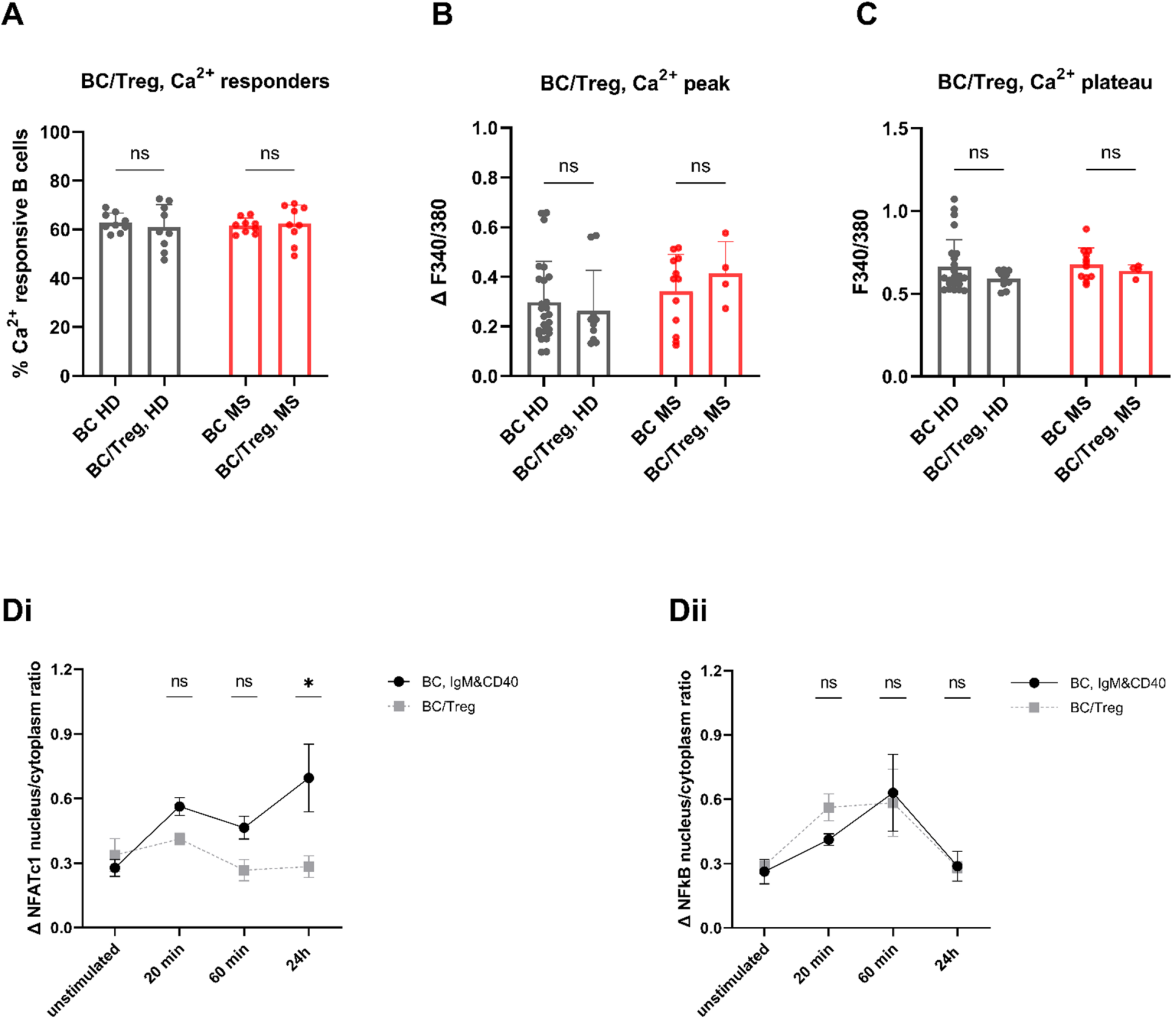
Ca^**2+**^ influx and NFATc1/NF-ĸB activation in anti-IgM/anti-CD40-stimulated B cells cocultured with Tregs.** A-C** Single-cell live Ca^2+^ imaging of B cells obtained from five HD in monoculture and in coculture with autologous Tregs showed that the presence of Tregs did not alter the percentage of responding B cells (**A**), Ca^2+^ peak amplitudes (**B**), or plateau Fura-2 signals (**C**). Results are based on a minimum of three independent experiments with more than 50 cells per experiment. (**D**) Treg-mediated effect on NFATc1 and NF-ĸB activation in anti-IgM/anti-CD40-stimulated B cells. (**Di**) B cells cocultured with Tregs (“BC/Treg”; HD, *n* = 5) exhibited significantly reduced NFATc1 nuclear translocation after 24 h of stimulation compared with monocultured B cells (“BC, IgM&CD40”; HD, *n* = 5). (**Dii**) In contrast, NF-ĸB activation rates were not significantly affected by coculture with Tregs. Bar charts in (A-C) show means ± SD with individual data points. Comparisons between two groups were performed using Welch’s t-tests. Comparisons among more than two groups were conducted using one-way ANOVAs with Kruskal‒Wallis tests with Dunn’s post-hoc corrections. Data in (D) are presented as mean ± SEM; comparisons are made using two-way ANOVA with Tukey’s multiple comparisons tests. BC = B cells; dual = anti-IgM/anti-CD40 stimulation; ns = non-significant. *P < 0.05

To assess whether Tregs influence Ca^2+^ signaling in B cells, and whether this differs between MS patients and HD, we performed single-cell live Ca^2+^ imaging in B cells obtained from 12 MS patients (median age 32 years; range 27–42; 9 female) and 25 age- and sex-matched HD (median age 30 years, range 23–37; 15 female). B cells were cocultured with autologous, anti-CD3/anti-CD28-stimulated Tregs at a 1:1 ratio and stimulated with anti-IgM/anti-CD40. Compared with monocultured, stimulated B cells, the presence of Tregs had no significant impact on the percentage of Ca^2+^ responsive B cells in either group (Fig. 9A). Similarly, the presence of Tregs had no impact on the Ca^2+^ peak amplitude or Ca^2+^ plateau phase of B cells in either HD- or MS-derived B cells (Figs. 9B, C). These results suggest that Tregs do not interfere with early BCR/CD40-induced Ca^2+^ signaling events in B cells.

#### Tregs suppress NFATc1 but not NF-ĸB in B cells

We next investigated whether Tregs affect downstream signaling pathways following BCR/CD40 stimulation. In B cells obtained from five HD, we analyzed nuclear translocation of NFATc1 and NF-ĸB at 20 min, 60 min, and 24 h after stimulation, comparing monocultured B cells with those cocultured with autologous, anti-CD3/anti-CD28-stimulated Tregs (1:1 ratio). Coculture with Tregs significantly reduced NFATc1 nuclear translocation (20 min: mean ΔNFATc1 nucleus/cytoplasm ratio 0.56, range 0.44–0.68; 60 min: mean 0.46, range 0.34–0.59; 24 h: mean 0.70, range 0.31–1.16), compared with monocultured B cells (20 min: mean 0.41, range 0.36–0.49, *P* = 0.016; 60 min: mean 0.27%, range 0.18–0.46, *P* = 0.029; 24 h: mean 0.28, range 0.26–0.38, *P* = 0.06; Fig. 9Di). In contrast, NF-ĸB nuclear translocation in stimulated B cells (20 min: mean 0.41, range 0.36–0.49; 60 min: mean 0.63%, range 0.26–1.16; 24 h: mean 0.29, range 0.13–0.53 [ΔNFATc1 nucleus/cytoplasm ratio]) was not significantly reduced by Treg coculture (20 min: mean 0.56, range 0.41–0.79, *P* = 0.012; 60 min: mean 0.58, range 0.13–0.84, *P* = 0.853; 24 h: mean 0.28, range 0.23–0.34, *P* = 0.951; Fig. 9Dii). These findings support the notion that Tregs selectively inhibit the Ca^2+^-dependent calcineurin–NFAT pathway in B cells, while sparing the Ca^2+^-independent NF-kB signaling axis.

## Discussion

### Summary of main findings

We show that human Tregs suppress BCR-stimulated B cells in vitro. The Treg-mediated inhibition was dose-dependent, Treg-specific, at least partially dependent on cell contact and affected proliferative responses, IL-6 secretion, and nuclear translocation of NFATc1.

As a key finding, Tregs obtained from MS patients displayed significantly lower suppressive effects on B cell proliferation and IL-6 secretion than HD-derived Tregs. Notably, data from crisscross experiments suggest that the MS-associated impaired Treg function towards B cells is paralleled by a reduced ability of MS-derived B cells to respond to suppressive Treg signals.

### Context with previous work on Treg-B cell interaction

A growing body of work has established that FoxP3⁺ regulatory T cells, including follicular regulatory T (Tfr) cells, are key controllers of B cell immunity in experimental models. Disruption or depletion of FoxP3⁺ T cells led to exaggerated GC responses, increased serum immunoglobulin levels and accumulation of autoreactive B cells, underscoring their importance for humoral tolerance [13, 14, 53]. At the cellular level, Tregs can directly inhibit B cell proliferation and antibody production through contact-dependent pathways, including granzyme- or TGF-β-mediated killing or functional silencing of activated B cells, and by limiting the help provided by T follicular helper cells [4, 5, 13, 54]. In addition, Tregs exploit immune-checkpoint pathways: CTLA-4 expression allows them to down-regulate costimulatory ligands such as CD80 and CD86 on B cells, while PD-1-ligand interactions can directly restrain PD-1⁺ autoreactive B cells [7]. More recently, Tregs have also been shown to modulate the antigen-presenting function of B cells in vivo, for example by limiting B cell-driven priming of T cell responses during infection [8].

Against this background, our data complement important insights into the B cell-Treg interaction in humans and add to the emerging picture by showing that, in MS, both the Treg-mediated suppressive axis and the responsiveness of B cells to such regulation are perturbed, which may contribute to the dysregulated humoral immunity characteristic of this disease.

### Possible mechanisms

Although the precise molecular mechanisms underlying Treg-mediated suppression of B cells in the experimental setup presented here remain to be elucidated, our findings suggest that suppression is unlikely to involve BCR-induced Ca^2+^ signals. However, Tregs did reduce the percentage of B cells entering the cell cycle and IL-6 secretion. Furthermore, Treg coculture significantly suppressed nuclear translocation of NFATc1, but not NF-κB, in BCR-stimulated B cells, suggesting that Tregs preferentially interfere with signaling steps downstream of Ca^2+^ influx, likely with the activation of the calcineurin–NFAT pathway [55, 56], leaving proximal NF-κB-dependent signaling largely intact.

Interestingly, we observed a trend toward increased Ca^2+^ responses in MS-derived B cells, particularly among the more responsive fraction of B cells. Of further note, preliminary data also suggest that CD27^−^ naïve B cells may exhibit higher Ca^2+^ responses than CD27^+^ memory B cells (Supplementary Fig. 7). These findings point toward a possible implication of dysregulated BCR signaling in MS, which was previously reported for several autoimmune diseases, including SLE, RA, and common variable immunodeficiency [57–61], thereby underscoring the need for further investigations into Ca^2+^ signaling in distinct B cell subpopulations. Moreover, Tregs (i) did not measurably increase apoptosis or cell death in B cells, (ii) left the expression of APC markers HLA-DR, CD80 and CD86 relatively intact and (iii) reduced IL-6 production per viable B cells, suggesting a Treg-mediated functional downregulation rather than deletion of activated B cells.

Mechanistic considerations that integrate our observations involve the Treg-mediated increase in intracellular cyclic AMP (cAMP) or adenosine in responder B cells, which has been shown to inhibit calcineurin activity without affecting calcium entry [62, 63]. In contrast, NF-κB activation relies on diacylglycerol (DAG)/protein kinase C (PKC)- and IκB kinase (IKK)-dependent pathways, which are largely independent of calcium–calcineurin signaling and may therefore be less susceptible to Treg-mediated suppression [64]. The more pronounced inhibition of NFATc1 compared with NF-κB may reflect a targeted strategy by Treg to attenuate B cell activation and proliferation without broadly suppressing inflammatory signaling.

Other mechanisms of Treg-induced B cell suppression that are compatible with our data include Treg-derived suppressive cytokines (e.g., IL-10) and inhibitory receptor pathways (e.g., PD-1, CTLA-4), which are known to dampen NFAT-driven transcription and cell-cycle entry without completely blocking initial activation signals [65–69]. Metabolic competition and altered metabolic programming [70–72] may also play a role by limiting the ability of B cells to sustain proliferation and cytokine output. Future work should clarify whether these pathways also extend to the interaction of Tregs with B cells in humans.

Preliminary bulk RNA-seq data identified a possible role in B cell inhibition of a number of candidate genes with known roles in activation and function of B cells (see Supplementary Fig. 8 for details).

### Strengths and limitations

We count the use of primary human biosamples, the inclusion of both healthy donors and patients with multiple sclerosis, the integration of complementary functional and signaling read-outs (Ca²⁺ responses, NFATc1/NF-κB nuclear translocation, proliferation, IL-6 production, apoptosis, and APC-marker expression), and the crisscross coculture design that helps to disentangle Treg-intrinsic defects from B cell-intrinsic resistance, among the strengths of this study. Limitations of our present study are sample size in the “mechanistic” sub-studies (IL-6, Annexin V, APC-markers) and the absence of data on the expression of immune checkpoints on Tregs and B cells. Future studies should in addition analyze B cell subsets, as the present study focused on total B cells and since functional differences between specific subpopulations cannot be ruled out. Moreover, B cell subset distribution differs markedly between HD and MS patients. In MS, class-switched and non-switched memory B cells migrate preferentially to the central nervous system, while naïve, CD27^−^ B cells are expanded in the peripheral blood [73–76], complicating direct comparisons of bulk B cell responses between groups. This should be considered in subsequent studies that should systematically incorporate B cell phenotyping (e.g. naïve, memory, double-negative, age-associated B cells) and assess subset-specific responsiveness to Tregs. Given the relatively early disease stage and low disability of the present cohort, and the expected initiation of disease-modifying therapy in many patients during follow-up, this study was not suited to determine whether impaired Treg-mediated suppression of B cells is associated with long-term disease activity or prognosis.

A further potential limitation of the present study is that we exclusively used combined anti-IgM/anti-CD40 stimulation to activate B cells in vitro. Numerous studies show that simultaneous BCR and CD40 binding is vital for B cell activation, metabolism and proliferation, making it a suitable model for investigating signaling responses and a robust platform for examining Tregs [26, 43–46, 77–80]. Here, stimulation with anti-IgM/anti-CD40 led to an increase in Ca²⁺ influx, IL-6 production, nuclear translocation of NFATc1 and NF-κB, expression of APC markers, and proliferation of HD B cells. However, further studies that utilize various B cell stimuli (CD40L, CpG, etc.) are of interest to assess if and how different modes of B cell activation (T cell-dependent versus T cell-independent pathways) affects Treg-mediated suppression.

Taken together, our data obtained in humans are consistent with a primarily post-receptor, NFAT-linked mechanism, but, considering the aforementioned limitations, the precise molecular pathways by which Tregs suppress B cell proliferation and IL-6 production remain to be fully elucidated.

## Conclusion

In sum, our data suggest that Tregs modulate B cell activation downstream of proximal BCR/CD40 signaling: early Ca²⁺ responses and NF-κB activation were largely preserved, whereas NFATc1 nuclear accumulation, Ki-67 expression and IL-6 production were disproportionately reduced compared with NF-κB activation, with no significant increase in late apoptosis and only modest effects on APC marker expression under the conditions tested. This pattern is compatible with mechanisms such as cAMP/adenosine-dependent suppression, inhibitory immune checkpoint pathways, Treg-derived cytokines, metabolic competition and altered metabolic programming. The finding that both Tregs from MS patients and MS B cells themselves are less responsive to this regulation supports a model in which impaired Treg function and B cell-intrinsic resistance jointly contribute to dysregulated humoral immunity in MS. Future studies dissecting cAMP/adenosine pathways, immune checkpoint engagement and metabolic programs in defined B cell subsets will be required to delineate these mechanisms in more detail.

## Supplementary Information

Supplementary Material 1.

## Data Availability

The datasets generated and/or analyzed during the current study are not publicly available but can be obtained from the corresponding author upon reasonable request.

## Abbreviations

BCR: B cell receptor
Ca²⁺: Calcium
cAMP: Cyclic adenosine monophosphate
DAG: Diacylglycerol
CD: Cluster of differentiation
EDSS: Expanded disability status scale
FoxP3: Transcription factor forkhead box protein 3
Fura-2: Fura-2-acetoxymethyl ester
HD: Healthy donors
IgM: Immunoglobulin M
IL: Interleukin
Ki-67: Marker of proliferation Kiel 67
mAbs: Monoclonal antibodies
MS: Multiple sclerosis
NFATc1: Nuclear factor of activated T cells, cytoplasmic 1
NF-κB: Nuclear factor ‘kappa-light-chain-enhancer’ of activated B cells
PBMCs: Peripheral blood mononuclear cells
PKC: Protein kinase C
RA: Rheumatoid arthritis
SLE: Systemic lupus erythematosus
TCR: T cell receptor
TGF-β: Transforming growth factor beta
TLR: Toll-like receptor
TNF-α: Tumor necrosis factor alpha
Tcons: Conventional T cells
Tregs: Regulatory T cells
