# Supplementary material for: Impaired suppressive effect of FoxP3 regulatory T cells on B cells in multiple sclerosis
Source: J Neuroinflammation. 2026 May 2;23:157. doi: 10.1186/s12974-026-03776-5 (PMC13179635; doi:10.1186/s12974-026-03776-5)
Supplement: Supplementary file 1 — Supplementary Material 1. [file 12974_2026_3776_MOESM1_ESM.docx]

**Supplement**

| **Antibodies** | **Vendor** | **Catalog No.** | **RRID** |
| --- | --- | --- | --- |
| **Flow Cytometry** |  |  |  |
| CD4-FITC | Miltenyi Biotec, Bergisch Gladbach, Germany | 130-113-815 | AB_2726332 |
| CD19-PE | BD Pharmingen, Heidelberg, Germany | 555413 | AB_395813 |
| CD19-Alexa Fluor 488 | BD Pharmingen, Heidelberg, Germany | 557697 | AB_396806 |
| CD20-PerCP | BD Pharmingen, Heidelberg, Germany | 345794 | AB_2868820 |
| CD25-FITC | Agilent Technologies, Waldbronn, Germany | F0801 | AB_2125618 |
| CD25-PE | BD Pharmingen, Heidelberg, Germany | 341011 | AB_2783790 |
| CD27-PE | BD Pharmingen, Heidelberg, Germany | 555441 | AB_395834 |
| CD80-APC | Thermo Fisher Scientific, Schwerte, Germany | 17-0809-42 | AB_2802217 |
| CD86-APC | Thermo Fisher Scientific, Schwerte, Germany | 17-0869-42 | AB_2802219 |
| Annexin V-APC | BD Pharmingen, Heidelberg, Germany | 550475 | AB_2868885 |
| FoxP3-APC | Thermo Fisher Scientific, Schwerte, Germany | 17-4776-42 | AB_1603280 |
| HLA-DR-APC | Thermo Fisher Scientific, Schwerte, Germany | MHLDR05 | AB_10374598 |
| Ki-67-APC | Thermo Fisher Scientific, Schwerte, Germany | 17-5699-42 | AB_2573218 |
| **Ca^2+^ Imaging** | | | |
| CD20 PerCP-Cy5.5 | BD Pharmingen, Heidelberg, Germany | 340954 | AB_400195 |
| CD4-Pacific Orange | Thermo Fisher Scientific, Schwerte, Germany | 79-0049-42 | AB_2815351 |
| CD25-PE | Thermo Fisher Scientific, Schwerte, Germany | R0811012 | AB_579546 |
| **NFATc1/NF-kB Microscopy** | | | |
| CD20 | Thermo Fisher Scientific, Schwerte Germany | PA5-16701 | AB_10980806 |
| CD3 | Dako, Agilent Technologies, Santa Clara, CA, USA | A0452 | AB_2335677 |
| NFATc1 | Santa Cruz Biotech. Heidelberg, Deutschland | sc-7294 | AB_2152503 |
| NFĸB | Santa Cruz Biotech. Heidelberg, Deutschland | sc-8008 | AB_628017 |
| anti-mouse-Cy3 | Jackson ImmunoResearch, Ely, UK | 115-165-166 | AB_2338692 |

**Supplementary Table 1. Comprehensive list of monoclonal antibodies utilized in the present study.**

RRID = Research Resource Identifier; na = not applicable

**
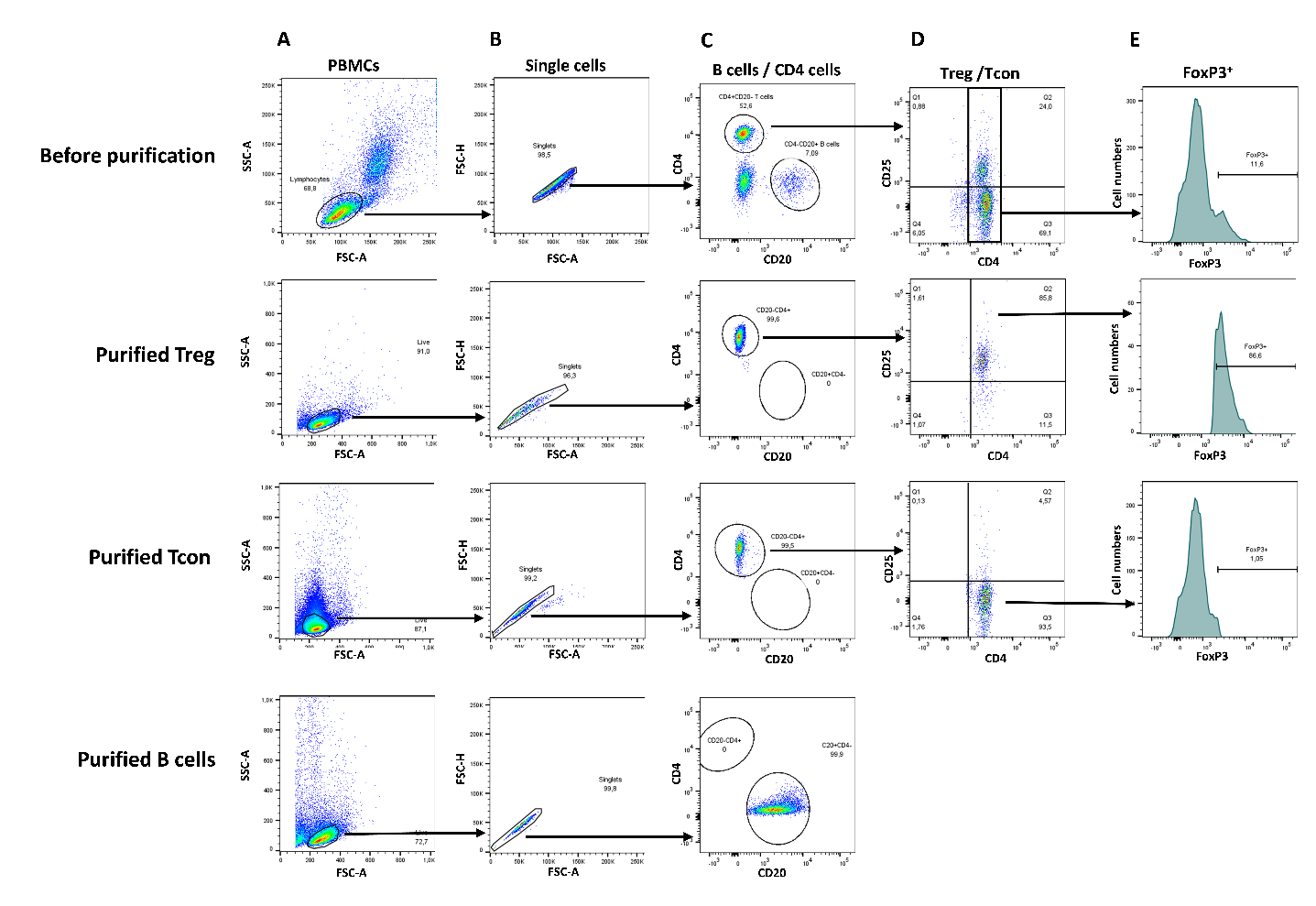
Supplementary Figure 1. Representative flow cytometry staining of B cell and T cell populations, before and following immunomagnetic purification from freshly isolated PBMCs.** To check purities, PBMCs and isolated cells were stained with fluorescence dye-labelled mAbs against CD4, CD20, CD25 and FoxP3 (mAbs used are listed in Supplementary Table 1). One representative sample each is shown for the gating strategy. **(A)** First, live PBMCs and purified cells were gated based on size and granularity using forward scatter (FSC-A) and side scatter (SSC-A) (live gate). **(B)** Next, cell doublets were out gated by FSC-A/FSC-H gating. **(C)** CD4^-^CD20^+^ total B cells and CD4^+^ T cells were then distinguished based on their CD20/CD4 expression. **(D)** CD4^+^CD20^-^CD25^high^ Tregs and CD4^+^CD20^-^CD25^low^ Tcons were identified in the CD4^+^ T cell gate based on their CD25 expression. **(E)** Tregs and Tcons were further analyzed for intracellular FoxP3 expression. Percentages indicate frequencies relative to the parent gate. Samples were acquired on a FACSCanto II flow cytometer, using CellQuest^TM^ software (BD Biosciences), and analyzed using FlowJo^TM^ (Version 10.7, Ashland, OR, USA).

**
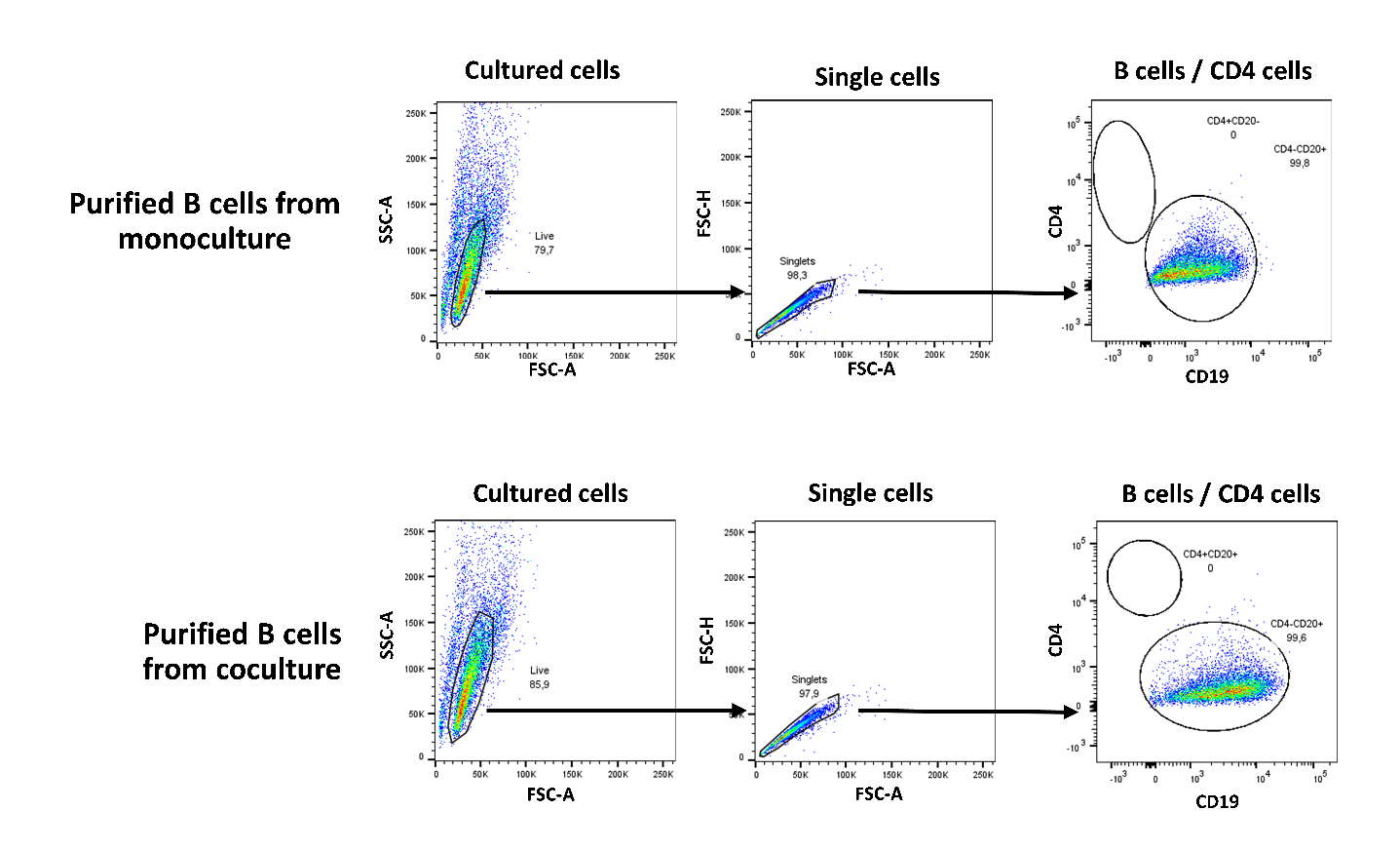
**

**Supplementary Figure 2. Representative flow cytometry staining of B cells following immunomagnetic purification after cell culture.** To isolate B cells following monoculture or coculture with Tregs, CD4^+^ cells were first depleted with CD4 MicroBeads, and total B cells were subsequently further purified by positive isolation using CD19 MicroBeads. To assess their purity, isolated B cells were double stained for CD19/CD4 to rule out T cell contamination (mAbs used are listed in Supplementary Table 1). One representative sample each is shown for the gating strategy. First, purified cells were gated based on size and granularity using forward scatter (FSC-A) and side scatter (SSC-A); cell doublets were then out gated by FSC-A/FSC-H gating and CD19^+^ total B cells and CD4^+^ T cells were finally distinguished based on their CD19/CD4 expression. Percentages indicate frequencies relative to the parent gate. Samples were acquired on a FACSCanto II flow cytometer, using CellQuest^TM^ software (BD Biosciences), and analyzed using FlowJo^TM^ (Version 10. 7, Ashland, OR, USA).

**
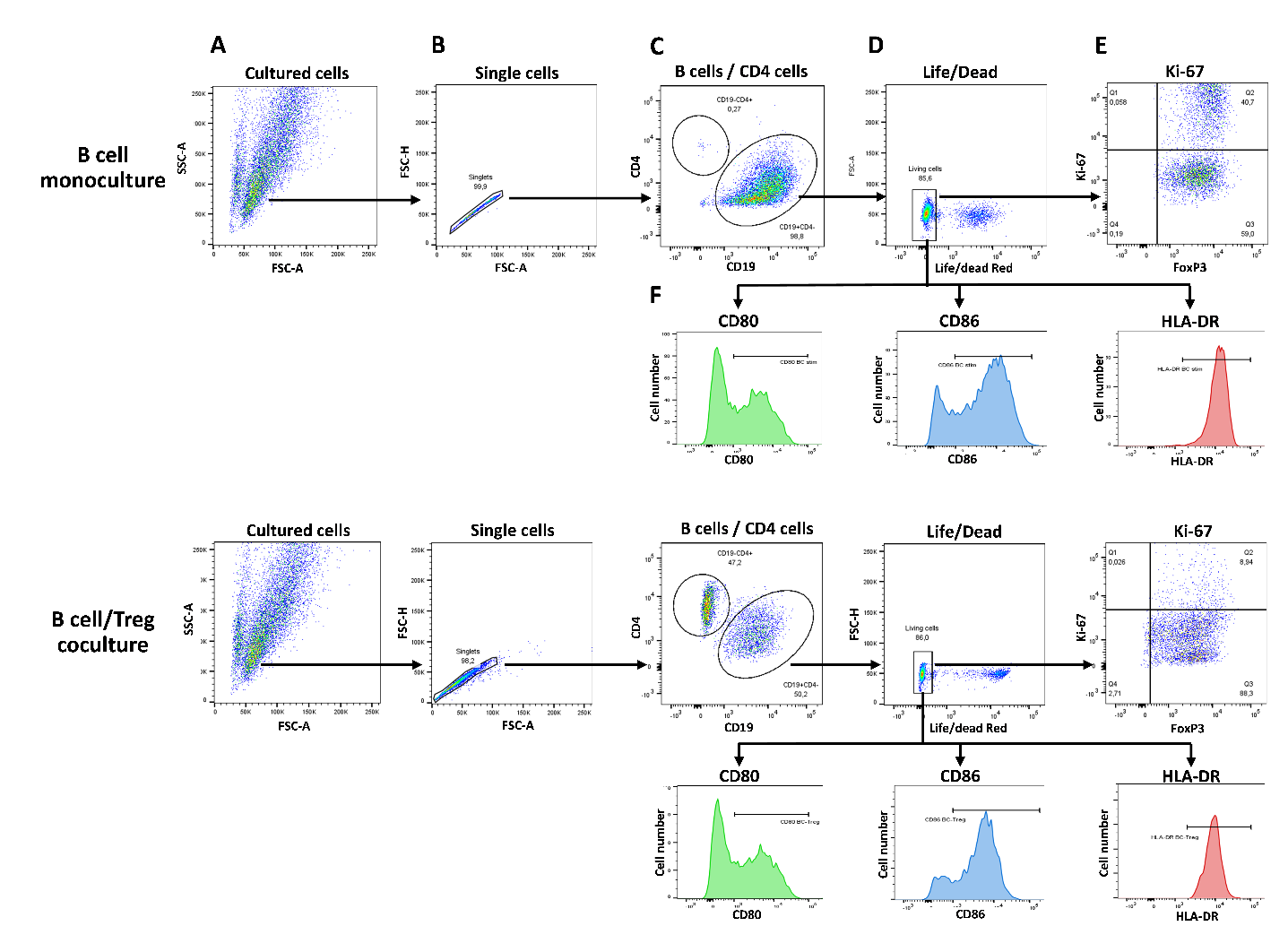
Supplementary Figure 3. Gating strategy for assessing Ki-67^+^ proliferating B cells, dead B cells and B cell APC markers by flow cytometry.** Following stimulation in B cell monoculture or B cell/Treg coculture, cultured cells were harvested and stained with mAbs against surface markers CD19 (total B cells) and CD4 (CD4^+^ T cells) to clearly distinguish between B cells and T cells (mAbs used are listed in Supplementary Table 1). One representative sample each is shown for the gating strategy.  **(A)** Stained cells were first gated based on size and granularity using forward scatter (FSC-A) and side scatter (SSC-A). **(B)** Cell doublets were then out gated by FSC-A/FSC-H gating. **(C)** CD4^-^CD19^+^ total B cells and CD4^+^CD19^-^ T cells were then distinguished based on their CD20/CD4 expression. **(D)** Dead/necrotic B cells were identified by free amine staining using the LIVE/DEAD™ Fixable Red Dead Cell Stain Kit (Thermo Fisher Scientific). (**E**) Proliferating B cells were identified in the living B cell gate based on intracellular expression of the nuclear proliferation marker Ki-67; APC markers by staining with mAbs, specific for HLA-DR, CD80 and CD86 (**F**). One representative sample is shown for the gating strategies used for monocultured B cells and B cell/Treg cocultures. Percentages indicate frequencies relative to the parent gate. Samples were acquired on a FACSCanto II flow cytometer, using CellQuest^TM^ software (BD Biosciences), and analyzed using FlowJo^TM^ (Version 10.7, Ashland, OR, USA).

**
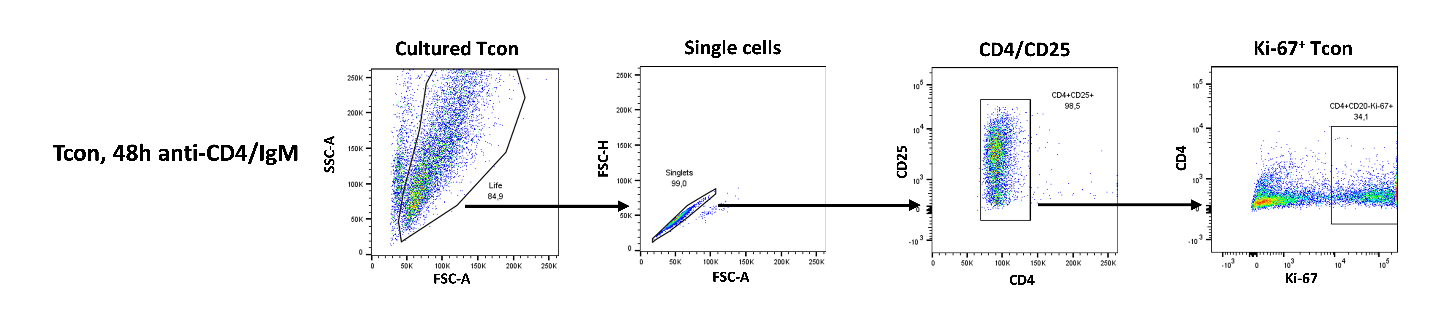
Supplementary Figure 4. Gating strategy for assessing successful T cell activation using the CD3/CD28 T cell activator by flow cytometry.** Following 48 hours of stimulation with 25 µl/ml CD3/CD28 T cell activator (Stemcell Technologies), Tcons were harvested and stained with fluorescence-labelled mAbs specific for CD4, CD25 and Ki-67 (mAbs used are listed in Supplementary Table 1). **(A)** Stained cells were first gated based on size and granularity by forward (FSC-A) and side scatter (SSC-A). **(B)** Cell doublets were then out gated by FSC-A/FSC-H gating. **(C)** Tcons were identified in the single cell gate based on their CD4/CD25 expression. **(D)** Finally, positive intracellular Ki-67 staining identified CD4^+^CD25^+^Ki-67^+^ proliferating Tcons. Percentages indicate frequencies relative to the parent gate. One representative sample is shown for the gating strategy. Samples were acquired on a FACSCanto II flow cytometer, using CellQuest^TM^ software (BD Biosciences), and analyzed using FlowJo^TM^ (Version 10.7, Ashland, OR, USA).

**
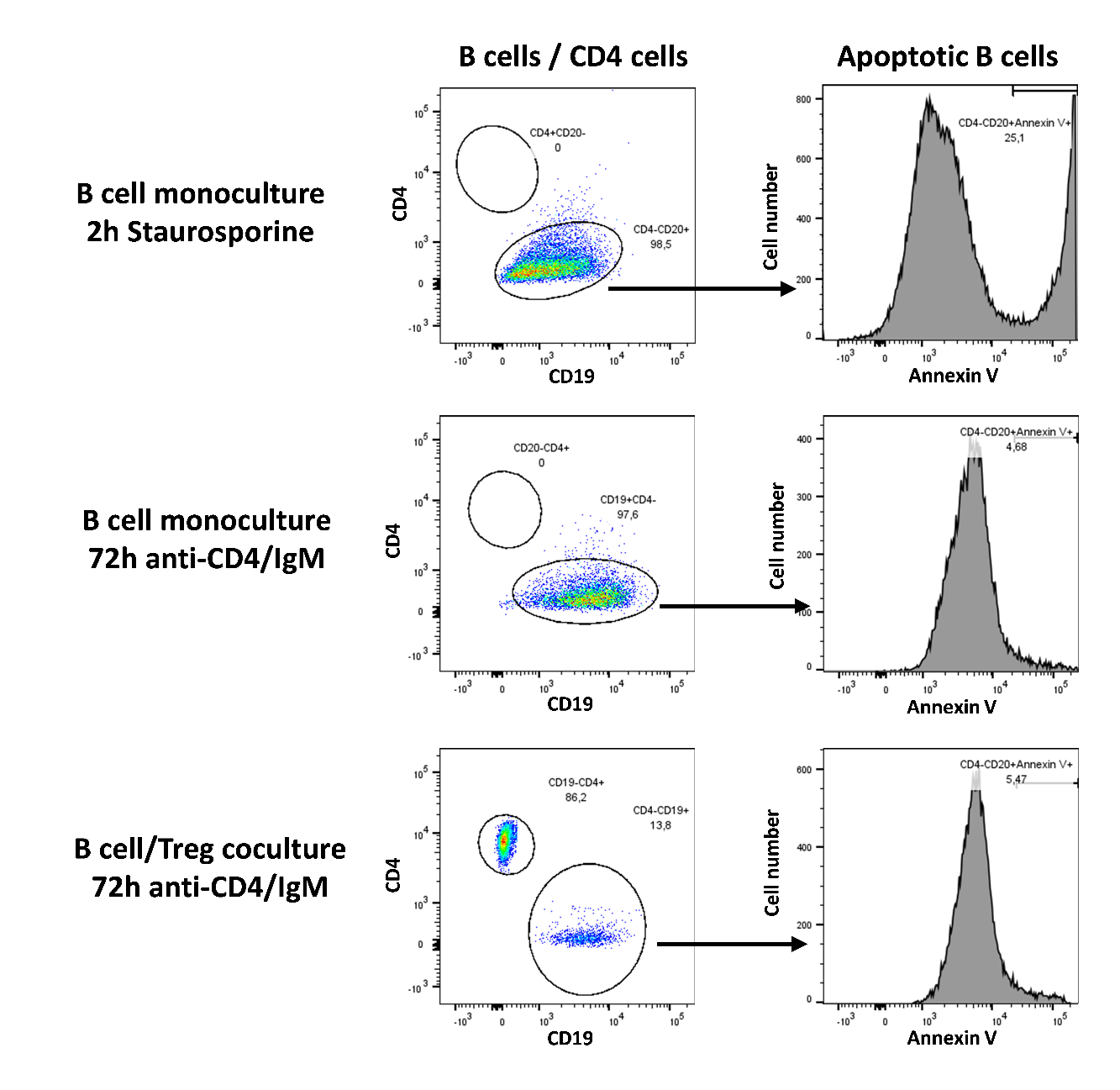
Supplementary Figure 5. Gating strategy for assessing the percentage of apoptotic B cells by flow cytometry.** Following stimulation in B cell monoculture or B cell/Treg coculture, cultured cells were harvested and then stained for CD4, CD19 and Annexin V. B cells, treated for 3 hours with 20 µM Staurosporine (Merck KGaA, Darmstadt, Germany) served as positive control. First, stained cells were first gated for size and granularity (FSC/SSC) and then for doublet exclusion (FSC-H/FSC-A) as shown in Supplementary Figures 1-3. Single cells were analyzed for CD4/CD19 expression to distinguish CD19^+^ total B cells and CD4^+^ T cells. Finally, Annexin V expression was determined in the CD4^-^CD19^+^ gate identifying apoptotic B cells. Percentages indicate frequencies relative to the parent gate. One representative sample is shown for the gating strategy. Samples were acquired on a FACSCanto II flow cytometer, using CellQuest^TM^ software (BD Biosciences), and analyzed using FlowJo^TM^ (Version 10.7, Ashland, OR, USA).

| **A** |  |  |  |  |  |  |  | **B** |  |  |
| --- | --- | --- | --- | --- | --- | --- | --- | --- | --- | --- |
| **Sample no.** | **B cells MS n = 7** | **B cells HD n = 7** | **Tregs MS n = 7** | **Tregs HD n = 7** | **Tcons MS n = 5** | **Tcons HD n = 5** |  | **Sample no.** | **B cells MS n = 6** | **B cells HD n = 6** |
| **#01** | 97.6% | 99.7% | 91.6% | 91.4% | 88.8% | 94.6% |  | **#01** | 99.6% | 98.4% |
| **#02** | 95.4% | 94.6% | 87.6% | 87.7% | 90.3% | 89.7% |  | **#02** | 99.7% | 97.8% |
| **#03** | 99.0% | 97.0% | 88.7% | 90.9% | 93.8% | 90.1% |  | **#03** | 96.8% | 95.6% |
| **#04** | 98.8% | 97.2% | 90.4% | 91.0% | 91.8% | 88.5% |  | **#04** | 98.2% | 99.0% |
| **#05** | 95.2% | 99.3% | 91.3% | 89.2% | 88.4% | 89.1% |  | **#05** | 97.0% | 98.4% |
| **#06** | 97.4% | 95.6% | 92.0% | 90.5% | na | na |  | **#06** | 99.7% | 99.2% |
| **#07** | 97.1% | 97.9% | 89.9% | 88.2% | na | na |  | **#07** | na | na |
| **mean** | **97.2%** | **97.3%** | **90.2%** | **89.8%** | **90.6%** | **90.4%** |  | **mean** | **98.5%** | **98.1%** |
| **min** | **95.2%** | **94.6%** | **87.6%** | **87.7%** | **88.4%** | **88.5%** |  | **min** | **96.8%** | **95.6%** |
| **max** | **99.0%** | **99.7%** | **92.0%** | **91.4%** | **93.8%** | **94.6%** |  | **max** | **99.7%** | **99.2%** |
| *P* |  | *0.901* |  | *0.660* |  | *0.885* |  | *P* |  | *0.587* |

**Supplementary Table 2. Purities of immunomagnetic isolated B and T cells.** Purities of total B cells, Tregs and Tcons freshly isolated from PBMCs of MS patients and HD. **(A)** and of total B cells isolated from cell cultures **(B)** were randomly checked by flow cytometry, revealing highly enriched cell populations for both MS patients and HD. Differences between purities of HD- and MS-derived cells were not statistically significant as determined by one-way ANOVA and Kruskal‒Wallis tests with Dunn’s post-hoc correction.

***
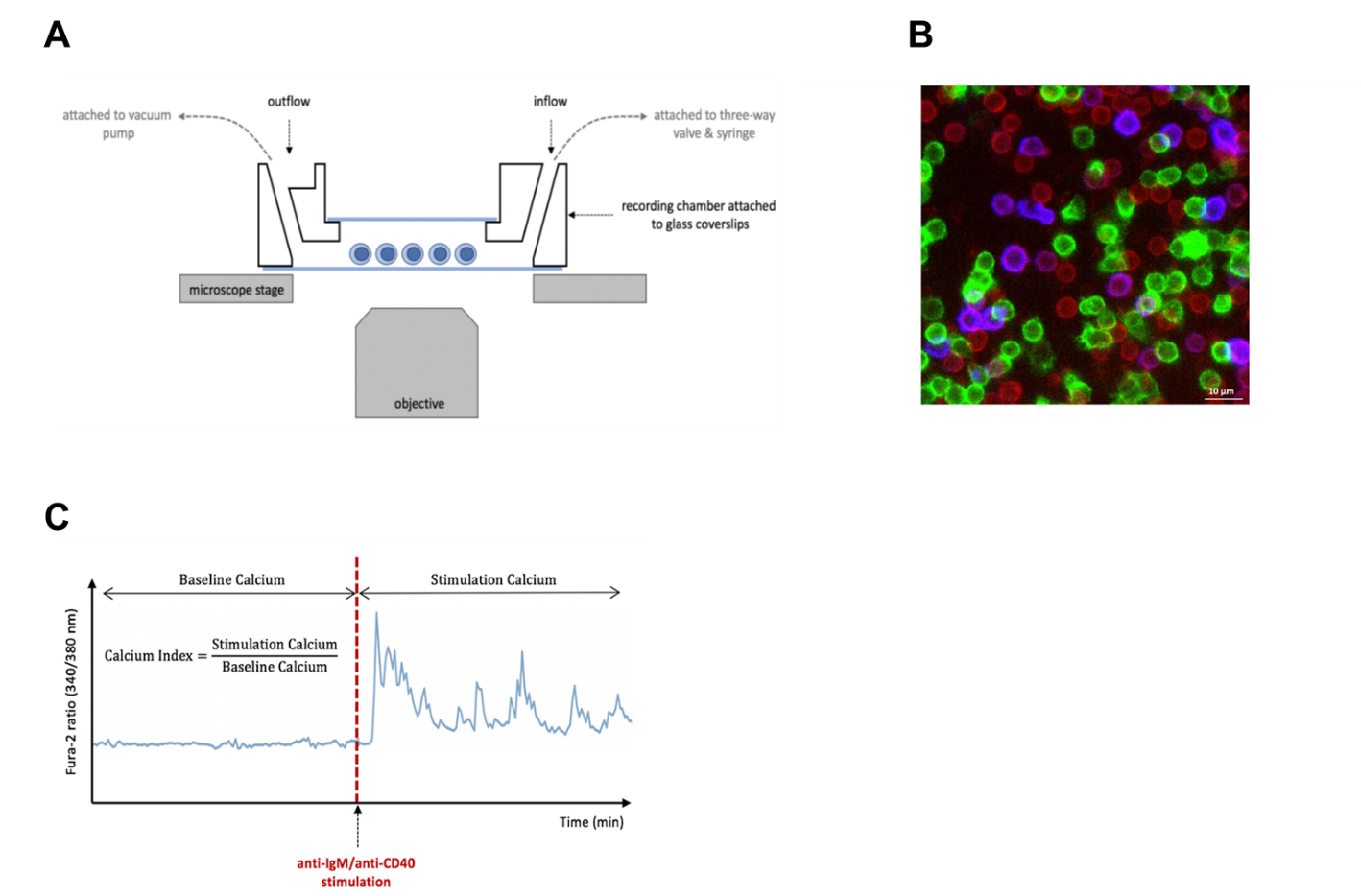
***

**Supplementary Figure 6. Live-cell calcium imaging experiments were performed using a custom-made recording chamber (A)**. Fura-2-loaded cells on a glass coverslip were attached to the bottom of the recording chamber, and a second coverslip was attached on top using silicone gel, forming a tightly sealed chamber. The bathing solution in the recording chamber could be exchanged during the experiment with the help of an inflow tube attached to a three-way valve and syringe, and an outflow tube attached to a vacuum pump (Schwarz et al. 2013*). **(B)** Following live-cell imaging experiments, cells were fixed and stained with mAbs against CD20, CD27 and CD4. B cells were identified based on their CD20^+^ expression. CD20^+^CD27^+^ B cells were identified as memory B cells (blue), and CD20^+^CD27^-^ cells as naïve B cells (red). Tregs were identified based on their CD4 expression (green).

*Schwarz A, Schumacher M, Pfaff D, Schumacher K, Jarius S, Balint B, et al. Fine-tuning of regulatory T cell function: the role of calcium signals and naive regulatory T cells for regulatory T cell deficiency in multiple sclerosis. J Immunol. 2013;190(10):4965-70.

**
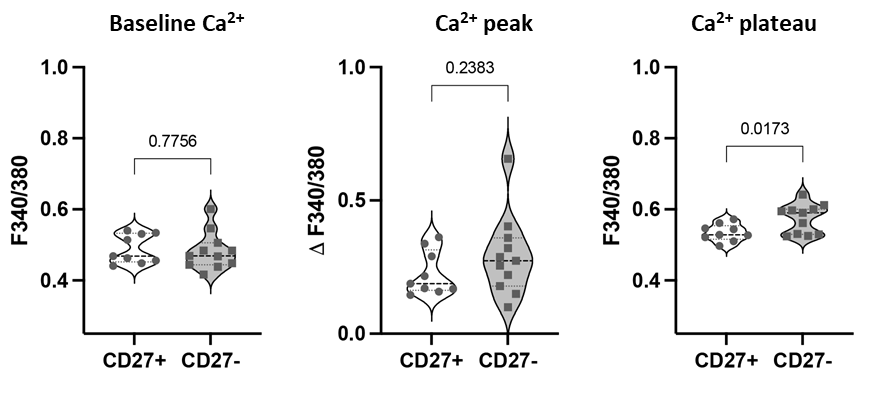
**

**Supplementary Figure 7. Preliminary analysis of Ca^2+^ responses in CD27^+^ memory and CD27^-^ naïve B cells.** In the B cell monoculture, there was a trend towards higher peak and plateau Fura-2 values in CD27^-^ naïve B cells compared to CD27^+^ memory B cells in response to stimulation with anti-IgM/anti-CD40. Violin plots represent the distribution of individual data points, as well as medians and 25th and 75th percentiles. P-values are based on Kruskal-Wallis tests with Dunn’s multiple comparisons.


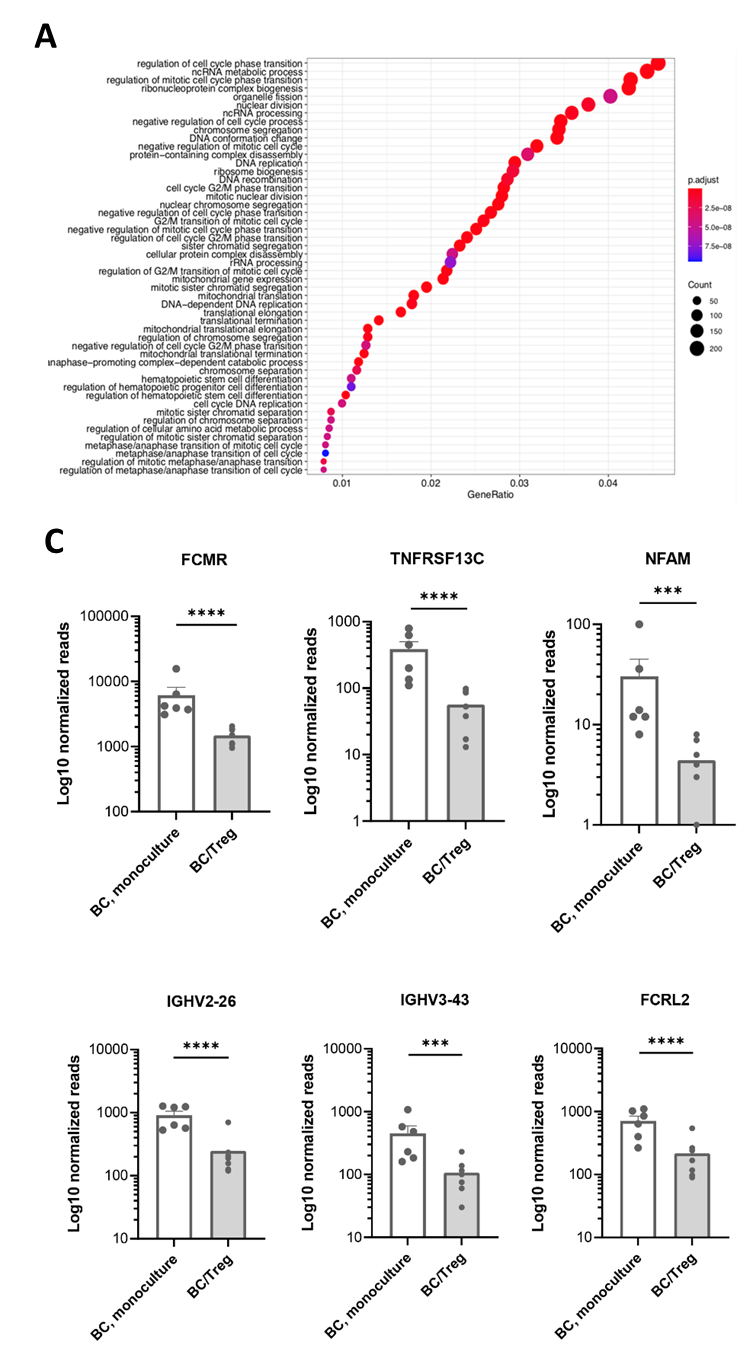


**Supplementary Figure 8. Treg-mediated effect on gene expression in human B cells.** As a preliminary exploratory analysis, bulk RNA-seq was performed on B cells. To this end, following stimulation in B cell monoculture or in B cell/Treg coculture, cultured cells were harvested, enriched for total B cells (Supplementary Figure 2, Supplementary Table 2B) and assessed in subsequent transcriptome analysis. In short, total RNA was extracted from B cells (Quick-RNA^TM^ Microprep Kit, Zymo Research Europe GmbH, Tübingen, Germany) and stored at –80°C until further processing. Bulk RNA sequencing and data analysis: Sample quality control and library preparation were performed externally by GENEWIZ Germany GmbH, Leipzig using the Clontech SMART-Seq v4 Ultra Low Input RNA kit. Libraries were pooled and loaded on the same lane of a NovaSeq flow cell for sequencing. Samples were sequenced on an Illumina HiSeq 2500 with a 2 × 150 bp paired-end configuration. The sequencing depth averaged around 29 million reads per sample. FASTQ files were trimmed for adapters and low-quality reads using Trim Galore! (https://www.bioinformatics.babraham.ac.uk /projects/trim_galore/) and aligned to the Ensembl GRCh38 reference genome (98th revision (<http://www.ensembl.org/Homo_sapiens/Info/Index>) using the pseudo aligner Salmon (1). Additional quality control assessments were performed using MultiQC (2). Aligned reads were further analyzed in RStudio (Version 1.4, Boston, MA, USA), using tximport and DESeq and with AnnotationHub and ensembldb to annotate gene symbols (3, 4). Sex was used as a regression variable. Plots were generated using ggplot2 (5). **(A)** B cells stimulated for 72 hours showed an enrichment in pathways associated with mitosis. Y-axis labels show GO pathway names, the x-axis represents the % genes that are differentially expressed in the associated pathway (gene ratio). Bubble size represents the number of genes enriched in the respective pathway; bubble color represents the p-value associated with the enrichment. GO dot plots were generated using R studio Version 1.4. **(B)** To assess the impact of Tregs on B cell gene expression, B cells isolated from a total of 13 HD were stimulated with anti-IgM/anti-CD40 and cultured for 72 hours, either alone or in coculture with autologous CD3/CD28-stimulated Treg at a 1:1 ratio. Following culture, B cells were repurified and subjected to differential gene expression analysis. Compared with B cells cultured alone (BC, monoculture; n = 6), coculture with Tregs (B cell:Treg, 72 hours; n = 7) led to significant downregulation of several B cell-specific genes, including *FCMR* (Fc fragment of IgM receptor), *TNFRSF13C* (encodes B cell-activating factor receptor), *NFAM1* (NFAT-Activating Protein with ITAM Motif 1), *IGHV2-26*, and *IGHV3-43* (immunoglobulin heavy variable genes; selected as representative examples of immunoglobulin heavy-chain variable region genes that showed consistent down-regulation in the presence of Tregs and have been implicated in B-cell activation in previous studies, and *FCRL2* (Fc Receptor-Like 2). The y-axis represents log_10_ normalized read counts. ***P<0.001; ****P<0.0001.

1. Patro R, Duggal G, Love MI, Irizarry RA, Kingsford C. Salmon provides fast and bias-aware quantification of transcript expression. Nature Methods. 2017;14(4):417-9.
2. Ewels P, Magnusson M, Lundin S, Kaller M. MultiQC: summarize analysis results for multiple tools and samples in a single report. Bioinformatics. 2016;32(19):3047-8.
3. Love MI, Huber W, Anders S. Moderated estimation of fold change and dispersion for RNA-seq data with DESeq2. Genome Biol. 2014;15(12):550.
4. Soneson C, Love MI, Robinson MD. Differential analyses for RNA-seq: transcript-level estimates improve gene-level inferences. F1000Res. 2015;4:1521.
5. Wickham H. ggplot2: Elegant Graphics for Data Analysis. Third Edition ed. New York: Springer; 2016.

| **Code** | **Gender** | **Age** | **Experimental group** | **Disease duration^1^** | **EDSS Score^2^** | **Disease activity^3^** | **Proliferation assay** | **Crisscross** | **Interleukin 6** | **Ca^2+^ Imaging** | **Apoptosis** | **Cell death** | **CD80, CD86, HLA-DR** | **NFATc, NFkB** | **Transwell assays** | **Dose dependence** | **Time course** | **Gene expression** |
| --- | --- | --- | --- | --- | --- | --- | --- | --- | --- | --- | --- | --- | --- | --- | --- | --- | --- | --- |
| MS01 | F | 40 | MS | 7 | 2,0 | rem | ✓ |  |  |  |  |  |  |  |  |  |  |  |
| MS02 | F | 29 | MS | 0 | 1,0 | rem |  |  |  | ✓ |  |  |  |  |  |  |  |  |
| MS03 | M | 30 | MS | 2 | 2,0 | rem | ✓ | ✓ |  |  |  |  |  |  |  |  |  |  |
| MS04 | F | 43 | MS | 0 | 1,0 | rem | ✓ |  |  |  |  |  |  |  |  |  |  |  |
| MS05 | F | 32 | MS | 0 | 2,0 | acute |  |  | ✓ |  |  |  |  |  |  |  |  |  |
| MS06 | F | 27 | MS | 1 | 1,5 | rem | ✓ |  |  |  |  |  |  |  |  |  |  |  |
| MS07 | F | 27 | MS | 0 | 1,0 | acute |  |  |  | ✓ |  |  |  |  |  |  |  |  |
| MS08 | M | 21 | MS | 2 | 2,0 | acute | ✓ |  |  |  |  |  |  |  |  |  |  |  |
| MS09 | F | 39 | MS | 4 | 3,0 | acute |  |  |  | ✓ |  |  |  |  |  |  |  |  |
| MS10 | F | 37 | MS | 11 | 2,0 | acute | ✓ |  |  |  |  |  |  |  |  |  |  |  |
| MS11 | M | 55 | MS | 0 | 2,0 | acute | ✓ | ✓ | ✓ |  |  |  |  |  |  |  |  |  |
| MS12 | F | 24 | MS | 0 | 2,0 | acute | ✓ | ✓ | ✓ |  |  |  |  |  |  |  |  |  |
| MS13 | F | 38 | MS | 0 | 1,0 | acute | ✓ | ✓ |  |  |  |  |  |  |  |  |  |  |
| MS14 | F | 32 | MS | nd | nd | rem | ✓ | ✓ |  |  |  |  |  |  |  |  |  |  |
| MS15 | F | 42 | MS | 2 | 2,0 | rem |  |  |  | ✓ |  |  |  |  |  |  |  |  |
| MS16 | F | 21 | MS | 2 | 2,5 | rem | ✓ | ✓ |  |  |  |  |  |  |  |  |  |  |
| MS17 | F | 32 | MS | 1 | 1,0 | acute |  |  |  | ✓ |  |  |  |  |  |  |  |  |
| MS18 | F | 28 | MS | 2 | 1,0 | acute | ✓ | ✓ |  |  |  |  |  |  |  |  |  |  |
| MS19 | F | 30 | MS | 3 | 3,5 | acute |  |  | ✓ |  |  |  |  |  |  |  |  |  |
| MS20 | M | 34 | MS | 3 | 2,0 | acute |  |  |  | ✓ |  |  |  |  |  |  |  |  |
| MS21 | M | 27 | MS | 3 | 2,0 | acute | ✓ |  |  |  |  |  |  |  |  |  |  |  |
| MS22 | M | 29 | MS | 0 | 1,0 | rem | ✓ | ✓ |  |  |  |  |  |  |  |  |  |  |
| MS23 | F | 23 | MS | 0 | 3,0 | acute | ✓ |  |  |  |  |  |  |  |  |  |  |  |
| MS24 | F | 43 | MS | 1 | 1,0 | rem |  |  | ✓ |  |  |  |  |  |  |  |  |  |
| MS25 | M | 29 | MS | 0 | 1,0 | acute |  |  |  | ✓ |  |  |  |  |  |  |  |  |
| MS26 | F | 41 | MS | 1 | 1,0 | acute | ✓ |  |  |  |  |  |  |  |  |  |  |  |
| MS27 | F | 35 | MS | 5 | 2,0 | acute |  |  |  | ✓ |  |  |  |  |  |  |  |  |
| MS28 | F | 26 | MS | 2 | 2,0 | rem | ✓ | ✓ |  |  |  |  |  |  |  |  |  |  |
| MS29 | F | 19 | MS | 0 | 1,0 | acute | ✓ |  |  |  |  |  |  |  |  |  |  |  |
| MS30 | F | 29 | MS | 2 | 2,0 | rem | ✓ | ✓ |  |  |  |  |  |  |  |  |  |  |
| MS31 | M | 33 | MS | 5 | 2,0 | acute |  |  |  | ✓ |  |  |  |  |  |  |  |  |
| MS32 | F | 38 | MS | 11 | 3,0 | acute | ✓ |  |  |  |  |  |  |  |  |  |  |  |
| MS33 | M | 34 | MS | nd | nd | rem | ✓ |  | ✓ |  |  |  |  |  |  |  |  |  |
| MS34 | F | 23 | MS | 2 | 2,0 | rem | ✓ |  |  |  |  |  |  |  |  |  |  |  |
| MS35 | F | 19 | MS | 2 | 2,0 | rem | ✓ |  |  |  |  |  |  |  |  |  |  |  |
| MS36 | F | 40 | MS | 0 | 2,0 | acute | ✓ | ✓ |  |  |  |  |  |  |  |  |  |  |
| MS37 | F | 42 | MS | 3 | 3,0 | acute | ✓ |  |  |  |  |  |  |  |  |  |  |  |
| MS38 | F | 32 | MS | 0 | 1,0 | acute |  |  |  | ✓ |  |  |  |  |  |  |  |  |
| MS39 | F | 27 | MS | 1 | 1,0 | rem |  |  |  | ✓ |  |  |  |  |  |  |  |  |
| MS40 | F | 27 | MS | 0 | 1,0 | acute |  |  |  | ✓ |  |  |  |  |  |  |  |  |
| HD01 | M | 26 | HD | na | na | na |  |  |  | ✓ |  |  |  |  |  |  |  |  |
| HD02 | M | 26 | HD | na | na | na |  |  |  | ✓ |  |  |  |  |  |  |  |  |
| HD03 | F | 29 | HD | na | na | na |  |  |  |  |  |  |  |  |  |  |  | ✓ |
| HD04 | M | 22 | HD | na | na | na | ✓ |  | ✓ |  |  |  |  | ✓ |  |  |  |  |
| HD05 | F | 22 | HD | na | na | na |  |  |  |  |  |  |  |  | ✓ |  |  |  |
| HD06 | F | 25 | HD | na | na | na |  |  |  |  |  |  |  |  |  |  | ✓ |  |
| HD07 | M | 25 | HD | na | na | na |  |  |  |  |  |  | ✓ |  |  |  |  |  |
| HD08 | F | 25 | HD | na | na | na |  | ✓ |  |  | ✓ |  |  |  |  |  |  |  |
| HD09 | M | 45 | HD | na | na | na | ✓ |  |  |  |  |  |  |  |  |  |  |  |
| HD10 | F | 27 | HD | na | na | na |  |  |  | ✓ |  |  |  |  |  |  |  |  |
| HD11 | F | 29 | HD | na | na | na |  | ✓ |  |  | ✓ |  |  |  |  |  |  |  |
| HD12 | M | 47 | HD | na | na | na | ✓ |  |  |  |  |  |  |  |  |  |  |  |
| HD13 | F | 33 | HD | na | na | na | ✓ |  |  |  |  |  |  |  |  |  |  |  |
| HD14 | F | 34 | HD | na | na | na |  |  |  | ✓ |  |  |  |  |  |  |  |  |
| HD15 | F | 46 | HD | na | na | na | ✓ |  |  |  |  |  |  | ✓ |  |  |  |  |
| HD16 | F | 55 | HD | na | na | na | ✓ | ✓ | ✓ |  |  |  |  |  |  |  |  |  |
| HD17 | F | 26 | HD | na | na | na | ✓ |  |  |  |  |  |  |  | ✓ |  |  |  |
| HD18 | F | 34 | HD | na | na | na |  |  |  |  |  |  |  |  |  |  |  | ✓ |
| HD19 | F | 28 | HD | na | na | na | ✓ |  |  |  |  |  |  |  |  |  |  |  |
| HD20 | M | 34 | HD | na | na | na |  |  |  | ✓ |  |  |  |  |  |  |  |  |
| HD21 | F | 25 | HD | na | na | na |  |  |  | ✓ |  |  |  |  |  |  |  |  |
| HD22 | F | 25 | HD | na | na | na |  |  |  | ✓ |  |  |  |  |  |  |  |  |
| HD23 | M | 21 | HD | na | na | na | ✓ |  |  |  |  |  |  | ✓ |  |  |  |  |
| HD24 | M | 56 | HD | na | na | na | ✓ |  |  |  |  |  |  |  |  |  |  |  |
| HD25 | F | 28 | HD | na | na | na |  |  |  |  |  |  |  |  |  |  |  | ✓ |
| HD26 | M | 24 | HD | na | na | na | ✓ |  |  |  |  |  |  | ✓ |  |  |  |  |
| HD27 | F | 36 | HD | na | na | na |  |  |  |  |  |  |  |  |  |  |  | ✓ |
| HD28 | F | 25 | HD | na | na | na | ✓ |  |  |  |  |  |  |  |  |  |  |  |
| HD29 | M | 22 | HD | na | na | na |  |  |  |  |  |  |  |  |  |  |  | ✓ |
| HD30 | M | 25 | HD | na | na | na | ✓ |  |  |  |  |  |  | ✓ |  |  |  |  |
| HD31 | F | 36 | HD | na | na | na |  |  |  | ✓ |  |  |  |  |  |  |  |  |
| HD32 | M | 29 | HD | na | na | na |  |  |  |  |  |  |  |  |  |  |  | ✓ |
| HD33 | F | 24 | HD | na | na | na | ✓ | ✓ |  |  |  |  |  |  |  |  |  |  |
| HD34 | M | 24 | HD | na | na | na |  |  |  | ✓ |  |  |  |  |  |  |  |  |
| HD35 | M | 40 | HD | na | na | na |  |  |  |  |  |  |  | ✓ |  |  |  |  |
| HD36 | F | 31 | HD | na | na | na |  |  |  | ✓ |  |  |  |  |  |  |  |  |
| HD37 | F | 23 | HD | na | na | na |  |  |  |  |  |  |  | ✓ |  |  |  |  |
| HD38 | M | 32 | HD | na | na | na |  |  |  |  |  |  |  |  | ✓ |  |  |  |
| HD39 | F | 38 | HD | na | na | na | ✓ | ✓ |  |  |  |  |  |  |  |  |  |  |
| HD40 | F | 38 | HD | na | na | na |  |  |  |  |  | ✓ |  |  |  | ✓ |  |  |
| HD41 | F | 34 | HD | na | na | na |  |  |  | ✓ |  |  |  |  |  |  |  |  |
| HD42 | F | 27 | HD | na | na | na |  |  |  |  |  | ✓ |  |  |  | ✓ |  |  |
| HD43 | F | 23 | HD | na | na | na |  |  |  |  |  |  |  |  |  |  |  | ✓ |
| HD44 | M | 25 | HD | na | na | na | ✓ |  |  |  |  |  |  |  |  |  |  |  |
| HD45 | M | 33 | HD | na | na | na | ✓ | ✓ |  |  |  |  |  |  |  |  |  |  |
| HD46 | F | 47 | HD | na | na | na |  |  |  |  |  | ✓ |  |  |  | ✓ |  |  |
| HD47 | M | 30 | HD | na | na | na | ✓ | ✓ |  |  |  |  |  |  |  |  |  |  |
| HD48 | M | 23 | HD | na | na | na |  |  |  | ✓ |  |  |  |  |  |  |  |  |
| HD49 | M | 24 | HD | na | na | na |  |  |  |  |  |  |  | ✓ |  |  |  |  |
| HD50 | F | 30 | HD | na | na | na |  |  |  |  |  | ✓ |  |  |  |  | ✓ |  |
| HD51 | F | 23 | HD | na | na | na |  |  |  |  |  |  |  |  |  |  | ✓ |  |
| HD52 | F | 37 | HD | na | na | na |  |  |  | ✓ |  |  |  |  |  |  |  |  |
| HD53 | F | 24 | HD | na | na | na |  |  |  |  |  |  |  | ✓ |  |  |  |  |
| HD54 | M | 34 | HD | na | na | na |  |  |  |  |  |  |  |  |  |  |  | ✓ |
| HD55 | F | 24 | HD | na | na | na | ✓ |  | ✓ |  |  |  |  | ✓ |  |  |  |  |
| HD56 | F | 37 | HD | na | na | na |  |  |  |  |  | ✓ |  |  |  |  | ✓ |  |
| HD57 | F | 50 | HD | na | na | na |  |  |  |  |  | ✓ |  |  |  |  | ✓ |  |
| HD58 | F | 35 | HD | na | na | na |  |  |  | ✓ |  |  |  |  |  |  |  |  |
| HD59 | F | 25 | HD | na | na | na |  |  |  |  |  |  |  |  | ✓ |  |  |  |
| HD60 | F | 45 | HD | na | na | na | ✓ |  |  |  |  |  |  |  |  |  |  |  |
| HD61 | M | 28 | HD | na | na | na |  |  |  | ✓ |  |  |  |  |  |  |  |  |
| HD62 | F | 31 | HD | na | na | na |  |  |  | ✓ |  |  |  |  |  |  |  |  |
| HD63 | M | 37 | HD | na | na | na |  |  |  | ✓ |  |  |  |  |  |  |  |  |
| HD64 | M | 26 | HD | na | na | na |  |  |  |  |  |  |  |  |  |  |  | ✓ |
| HD65 | F | 23 | HD | na | na | na |  |  |  | ✓ |  |  |  |  |  |  |  |  |
| HD66 | F | 57 | HD | na | na | na |  |  |  |  |  |  |  | ✓ |  |  |  |  |
| HD67 | F | 30 | HD | na | na | na |  |  |  | ✓ |  |  |  |  |  |  |  |  |
| HD68 | F | 35 | HD | na | na | na | ✓ |  | ✓ |  |  |  |  | ✓ |  |  |  |  |
| HD69 | F | 25 | HD | na | na | na |  |  |  |  |  |  |  |  |  |  |  | ✓ |
| HD70 | F | 29 | HD | na | na | na | ✓ |  |  |  |  |  |  |  | ✓ |  |  |  |
| HD71 | M | 32 | HD | na | na | na |  |  |  | ✓ |  |  |  |  |  |  |  |  |
| HD72 | F | 23 | HD | na | na | na |  |  |  |  |  |  |  | ✓ |  |  |  |  |
| HD73 | F | 32 | HD | na | na | na |  |  |  | ✓ |  |  |  |  |  |  |  |  |
| HD74 | F | 23 | HD | na | na | na |  |  |  |  |  |  |  | ✓ |  |  |  |  |
| HD75 | F | 33 | HD | na | na | na |  |  |  | ✓ |  |  |  |  |  |  |  |  |
| HD76 | F | 23 | HD | na | na | na | ✓ |  |  |  |  |  |  | ✓ |  |  |  |  |
| HD77 | F | 23 | HD | na | na | na | ✓ | ✓ |  |  |  |  |  |  |  |  |  |  |
| HD78 | F | 22 | HD | na | na | na |  |  |  |  |  |  |  |  | ✓ |  |  |  |
| HD79 | M | 33 | HD | na | na | na |  |  | ✓ |  |  |  |  |  |  |  |  |  |
| HD80 | F | 22 | HD | na | na | na |  |  |  |  |  |  |  |  |  |  |  | ✓ |
| HD81 | F | 22 | HD | na | na | na | ✓ |  |  |  |  |  |  |  |  |  |  |  |
| HD82 | F | 40 | HD | na | na | na | ✓ |  | ✓ |  |  |  |  |  |  |  |  |  |
| HD83 | F | 39 | HD | na | na | na | ✓ |  |  |  |  |  |  |  |  |  |  |  |
| HD84 | M | 49 | HD | na | na | na |  | ✓ |  |  | ✓ |  |  |  |  |  |  |  |
| HD85 | F | 27 | HD | na | na | na |  |  |  | ✓ |  |  |  |  |  |  |  |  |
| HD86 | M | 37 | HD | na | na | na |  |  |  |  |  |  |  |  |  |  |  | ✓ |
| HD87 | M | 28 | HD | na | na | na |  |  |  | ✓ |  |  |  |  |  |  |  |  |
| HD88 | M | 44 | HD | na | na | na |  |  |  |  |  |  |  | ✓ |  |  |  |  |
| HD89 | M | 23 | HD | na | na | na |  |  |  | ✓ |  |  |  |  |  |  |  |  |
| HD90 | F | 41 | HD | na | na | na |  |  |  |  |  |  |  | ✓ |  |  |  |  |
| HD91 | F | 31 | HD | na | na | na |  |  |  |  |  |  |  |  |  |  |  | ✓ |
| HD92 | M | 26 | HD | na | na | na |  | ✓ |  |  |  |  | ✓ |  |  |  |  |  |
| HD93 | F | 27 | HD | na | na | na |  |  |  |  |  |  |  | ✓ |  |  |  |  |
| HD94 | F | 41 | HD | na | na | na | ✓ |  |  |  |  |  |  |  |  |  |  |  |
| HD95 | F | 35 | HD | na | na | na |  |  |  |  |  |  | ✓ |  |  |  |  |  |
| HD96 | M | 44 | HD | na | na | na |  |  |  |  | ✓ |  | ✓ |  |  |  |  |  |
| HD97 | F | 56 | HD | na | na | na |  |  |  |  | ✓ |  | ✓ |  |  |  |  |  |
| HD98 | F | 32 | HD | na | na | na |  |  |  |  | ✓ |  | ✓ |  |  |  |  |  |

**Supplementary Table 3. Demographic and clinical characteristics of the 138 study subjects and their participation in the study experiments.** M = male, F = female, MS = multiple sclerosis, HD = healthy donor; 1 = years from disease onset to blood sampling; 2 = Expanded Disability Status Scale; 3 = Disease activity at time of blood sampling (acute = acute relapse/active disease stage; rem = clinical remission/ inactive disease stage); check marks indicate participation in an experiment; na = not applicable; nd = no data available.
